# Gold-Catalyzed Synthesis of Tetrazoles from Alkynes by C=C Bond Cleavage**

**DOI:** 10.1002/anie.201308076

**Published:** 2013-11-13

**Authors:** Morgane Gaydou, Antonio M Echavarren

**Affiliations:** Institute of Chemical Research of Catalonia (ICIQ)Av. Països Catalans 16, 43007 Tarragona (Spain); Departament de Química Analítica i Química Orgànica, Universitat Rovira i VirgiliC/Marcel⋅li Domingo s/n, 43007 Tarragona (Spain)

**Keywords:** azides, cycloadditions, gold, heterocycles, rearrangement

**Scheme 1:**
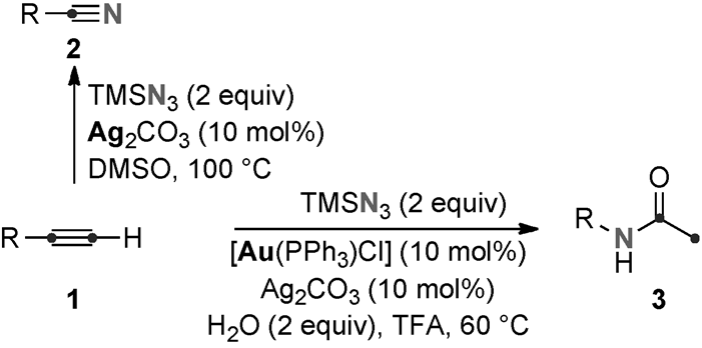
Synthesis of nitriles (2)[9] and carboxamines (3)[10] from alkynes (1) by aryl–alkyne C(sp^2^)=C(sp) bond cleavage. DMSO=dimethylsulfoxide, TFA=trifluoroacetic acid, TMS=trimethylsilyl.

Cycloadditions of azides with alkynes to form triazoles under thermal conditions (Huisgen cycloaddition)[1] or in the presence of copper [click reaction, copper-catalyzed azide–alkyne cycloaddition (CuAAC)][2,3] are reactions of fundamental importance in organic chemistry. Triazoles can also be obtained by means of ruthenium,[4] silver,[5] and iridium[6] catalysis, as well as by a zinc-mediated process.[7] In sharp contrast, very different reactivity has been observed in the reaction of terminal alkynes with TMSN_3_ in the presence of group 11 metal salts and complexes.[8] Thus, the group of Jiao recently made the remarkable observation that alkynes (**1**; R=alkyl, aryl, alkenyl) react with TMSN_3_ in the presence of Ag_2_CO_3_ as catalyst to form nitriles (**2**; Scheme 1).[9] The same group has reported the cleavage of the aryl**–**alkyne C(sp^2^)=C(sp) bond of alkynes (**1**) using [Au(PPh_3_)Cl] and AgCO_3_ in the presence of H_2_O and trifluoroacetic acid (TFA) to form carboxamides.[10]

**Scheme 2:**
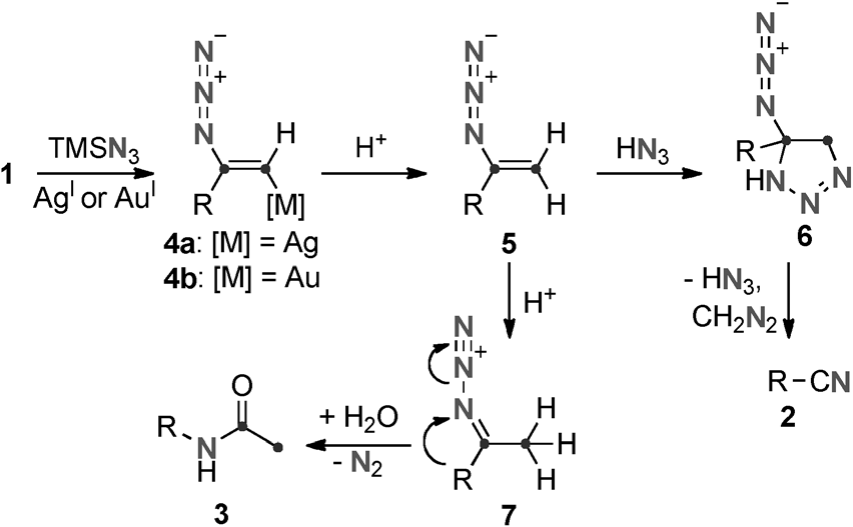
Mechanistic proposal for the formation of 2 and 3.[9,10]

The formation of nitriles (**2**)[9] and carboxamides (**3**)[10] was proposed to proceed by nucleophilic addition of azide to (η^2^-alkyne)metal complexes to form the intermediates **4 a**,**b**, with subsequent protonolysis to give the alkenyl azides **5** (Scheme 2). The nitriles **2** could then be produced by a 1,3-dipolar cycloaddition and subsequent fragmentation of **6**. In the presence of TFA, protonation of **5** would form **7**, which could evolve by a Schmidt rearrangement[11–13] to give the amides **3**. A somewhat related cleavage of triple bonds to form nitriles has been reported using TMSN_3_ and *N*-iodosuccinimide, and was proposed to proceed via 2-iodo-2*H*-azirines.[14]

**Scheme 3:**
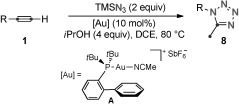
Synthesis of N-aryltetrazoles (8) from alkynes (1). DCE=1,2,-dichloroethane.

We now report that by using the JohnPhos/gold(I) catalyst **A**,[15] which allows performing reactions in the absence of Ag^I^, the *N*-aryltetrazoles **8** are obtained from **1** by C=C bond cleavage with the concomitant insertion of four nitrogen atoms (Scheme 3). In this transformation gold plays a dual role, first activating the alkyne towards nucleophilic attack and then generating the Brønsted acid required for the transformation of the alkenyl azide into the final tetrazole.

**Scheme 4:**
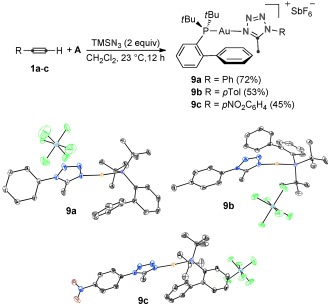
Formation of the tetrazole–gold(I) complexes 9 a–c and their X-ray crystal structures. For the ORTEP plots the thermal ellipsoids are shown at 50 %. Au yellow, F green, N blue, O red, P violet, Sb light blue.

We first studied the reaction of the aryl alkynes **1 a**–**c** with TMSN_3_[16] and complex **A** under stoichiometric conditions. Surprisingly, the reaction gave 5-methyl-1-aryl-1*H*-tetrazole–gold(I) complexes (**9 a**–**c**) as crystalline white solids, whose structures were determined by X-ray diffraction (Scheme 4).[17,18] The complex **9 a** was also obtained in 56 % yield by reaction of neutral [(JohnPhos)AuCl] with phenyl acetylene (**1 a**) and TMSN_3_ in the presence of AgSbF_6_.

We have provided evidence that the rate-determining step in certain catalytic reactions involving alkynes is the ligand substitution reaction between the complexes [Au(product)L]^+^ and the starting alkyne.[19] The isolation of stable gold(I) complexes (**9 a**–**c**) under stoichiometric conditions shows that in this case the development of a catalytic process for the synthesis of tetrazoles would be a challenging task, since this ligand substitution would be particularly slow. Thus, either no reaction or very poor yields of the tetrazole **8 d** were obtained with complex **A** in MeCN, CH_2_Cl_2,_ or toluene (Table 1, entries 1–4). Better results were obtained in 1,2-dichloroethane at 80 °C (Table 1, entries 5 and 6). In contrast, the related gold(I) catalysts **B** and **C**, and complexes **D**–**G** with NHC (N-heterocyclic carbene), phosphite, or less-bulky phosphine ligands led to poor results (Table 1, entries 9 and 16).

**Table 1:** Catalyst and solvent optimization for the formation of 8 b. 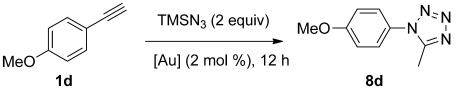

Entry	[Au]	Solvent	*T* [°C]	Yield [%][a]
1	**A**	MeCN	23	–[b]
2	**A**	MeCN	80	8
3	**A**	CH_2_Cl_2_	40	–[b]
4	**A**	toluene	110	9
5	**A**	DCE	80	40
6	**A**[c]	DCE	80	59
7[d]	**A**[c]	DCE	80	78–81
8	**A**	DCE	110	38
9	**B**	DCE	80	8
10	**C**	DCE	80	7
11	**D**	DCE	80	–[b]
12	**D′**	DCE	80	–[b]
13	**E**	DCE	80	–[b]
14	**F**	DCE	80	15
15	**G**	DCE	80	18
16	[Au(PPh_3_)Cl]/ Ag_2_CO_3_	DCE	80	–[b]

[a] Determined by NMR spectroscopy.

[b] No reaction.

[c] 10 mol % catalyst.

[d] *i*PrOH (4–10 equiv).
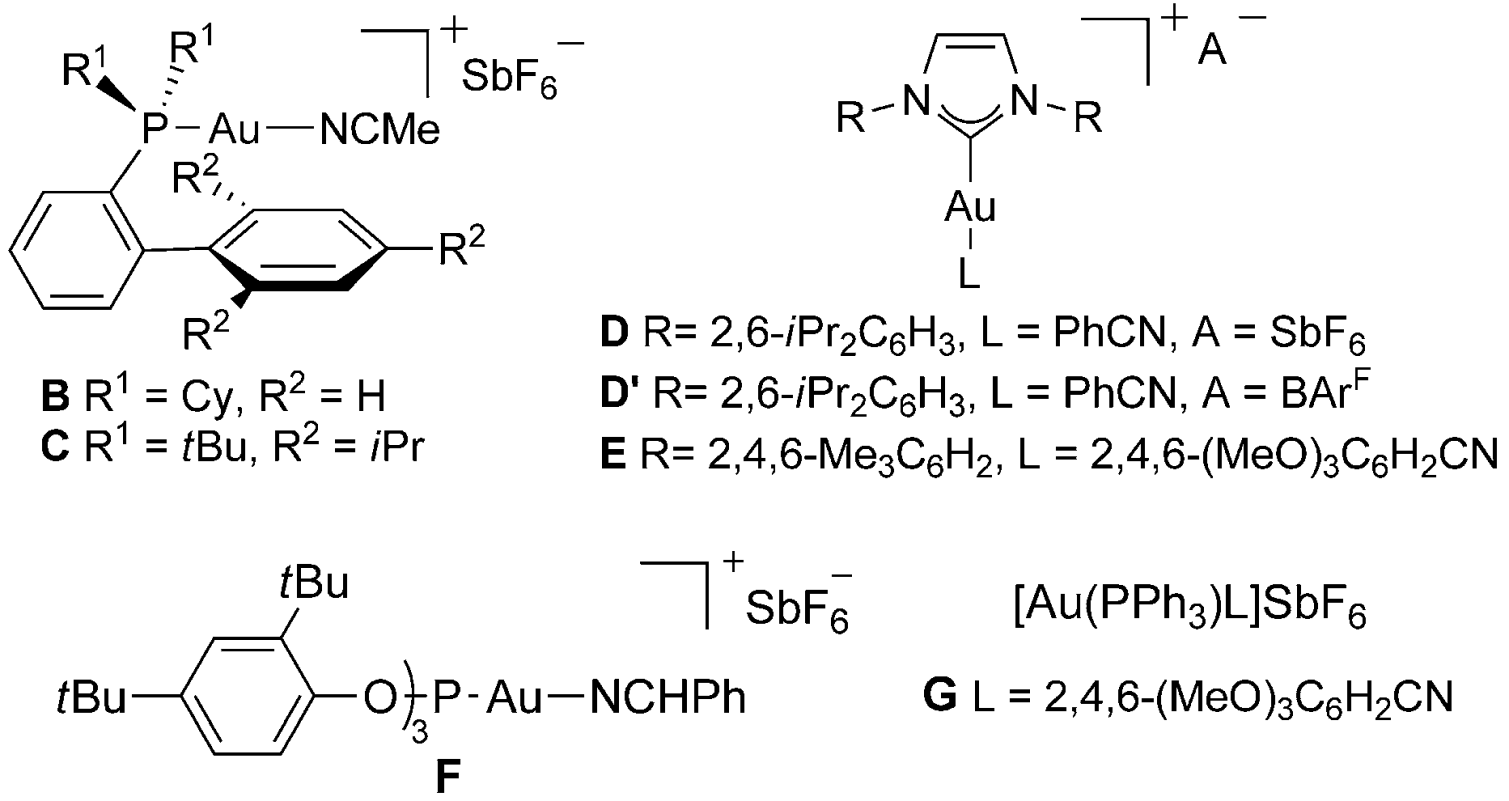

**Scheme 5:**
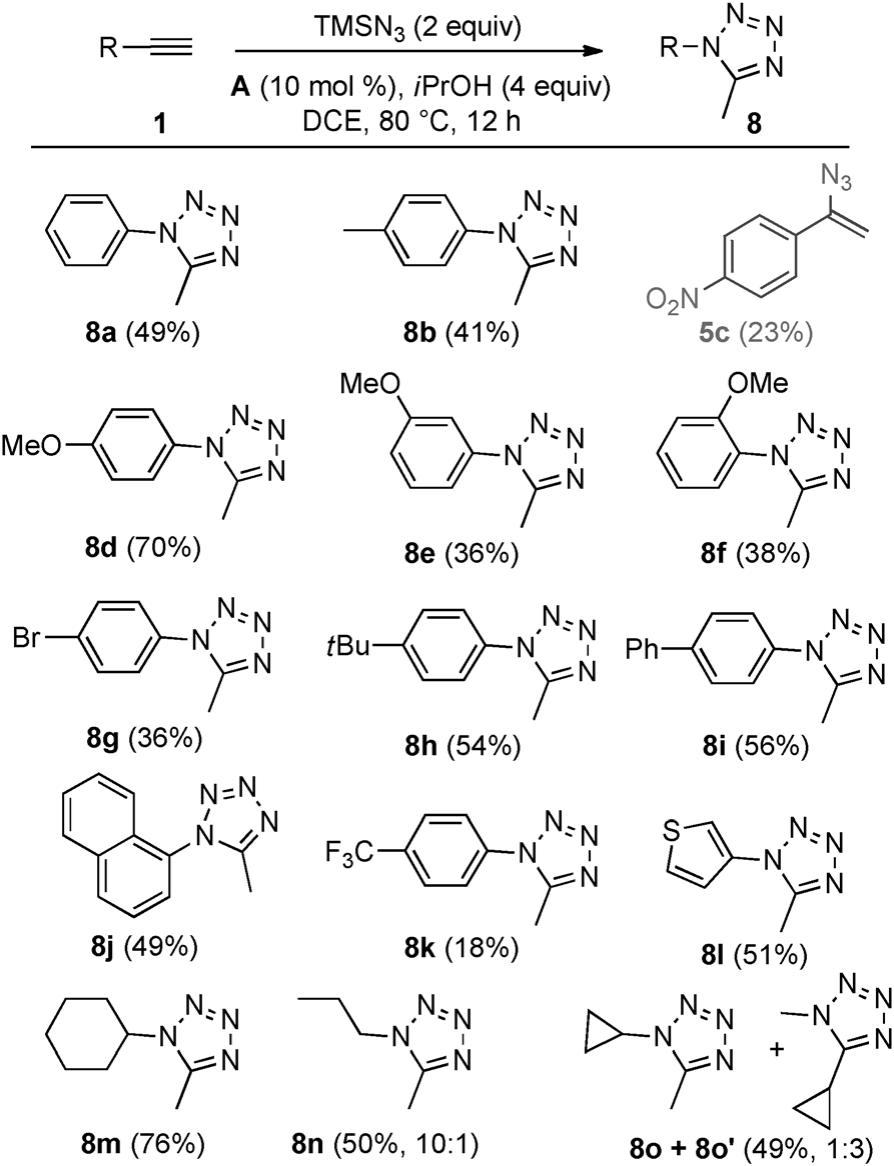
Gold(I)-catalyzed synthesis of tetrazoles from the aryl- and alkyl-substituted alkynes 1. Yields refer to isolated compounds.

A further improvement was achieved by performing the reaction in the presence of *i*PrOH (Table 1, entry 7). Under these reaction conditions, aryl-, heteroaryl-, and alkyl-substituted alkynes react with TMSN_3_ to give the corresponding tetrazoles **8** (Scheme 5). Lower yields of the tetrazoles **8 g** and **8 k** were obtained from employing aryl alkynes substituted with electron-withdrawing groups. In the case of *p*-nitrophenylacetylene (**1 c**), no tetrazole was formed and the alkenyl azide **5 c** was isolated instead (23 % yield). Diphenyl acetylene, having an internal alkyne, failed to give the corresponding tetrazole. Aliphatic alkynes also reacted to give tetrazoles (**8 m**–**o**). Interestingly, whereas cyclohexylacetylene provided **8 m** in good yield as the sole product, 1-pentyne gave **8 n** along with 1-methyl-5-propyl-1*H*-tetrazole (**8 n′**; 10:1 ratio) and cyclopropylacetylene gave **8 o** and **8 o′** (1:3 ratio).

**Scheme 6:**
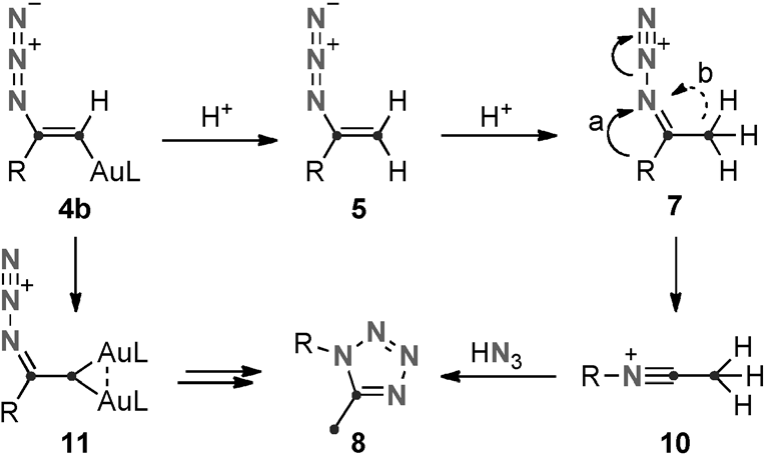
Mechanistic proposal for the formation of the tetrazoles 8 from 4 b.

All these results can be accommodated by a mechanism proceeding by reaction between a (η^2^-alkyne)gold(I) complex and HN_3_, formed in situ from TMSN_3_ and *i*PrOH, to give **4 b**, which undergoes protodeauration to give **5** (Scheme 6), and is in accordance with that proposed for the formation of nitriles and carboxamides.[9,10] Protonation of **5** would give the iminodiazonium cation **7**, which could evolve to form the nitrilium cation **10** by migration of R group (path a). Competitive migration of the methyl group (path b) explains the formation of regioisomers **8 n′** and **8 o′** in the reactions of 1-pentyne and cyclopropylacetylene. It is interesting that preferential migration of the methyl group has been observed in the Schmidt reaction of methyl cyclopropyl ketone in aqueous sulfuric acid at lower acid strengths.[12a] Finally, a formal 1,3-dipolar cycloaddition of HN_3_ to **10** would lead to **8**.[20,21] It is important to note that nitrilium cations **10** have been reported to give also triazolium salts by reaction of the initial azide addition product with a second nitrilium cation,[21b] a process that was not observed under these reaction conditions.

Although formation of digold(I) intermediates (**11**) by reaction of **4 b** with a second equivalent of a gold(I) complex cannot be entirely excluded,[22] the following experiments using (1-azidovinyl)benzene (**5 a**, R=Ph) as the substrate strongly suggest that the transformation of **4 b** into **7** is a Brønsted acid catalyzed reaction: 1) reaction of **5 a** with TMSN_3_ and *i*PrOH with **A** under the standard reaction conditions gave **8 a** (42 % yield by NMR); 2) in the absence of *i*PrOH, **5 a** gave **8 a** in only 12 % yield; 3) only traces of **8 a** were obtained in the absence of gold catalyst **A**; 4) replacing *i*PrOH and **A** by HOAc (2 equiv) led to **8 a** in 78 % yield.[23] Presumably, under the gold(I)-catalyzed conditions, the Brønsted acid [JohnPhosAu(*i*PrOH)]SbF_6_ is formed, which mediates the transformation of **4 b** into **7**.[24,25] Protonation by this acid could also facilitate the associative displacement of the tetrazole ligands by the incoming alkyne in **9** under catalytic conditions.

**Scheme 7:**
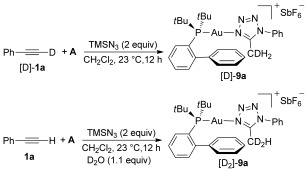
Deuterium-labeling experiments.

Acetophenones, which could have been formed by gold(I)-catalyzed hydration, were not detected in this reaction.[26,27] The proposed mechanism was further supported by additional results, including two labeling experiments. First, reaction of [D]-**1 a** led to [D]-**9 a**, with the deuterium labeling at the methyl group (Scheme 7). Additionally, when the reaction of **1 a**, TMSN_3_, and complex **A** was carried out in CH_2_Cl_2_ containing 1.1 equivalents of D_2_O, the deuterated complex [D_2_]-**9 a** was obtained.

Tetrazoles, which are important in medicinal chemistry and as energetic materials, have been obtained by 1,3-dipolar cycloaddition of azides with activated nitriles[28,29] and by cycloaddition of hydrazoic acid with the Ugi adducts generated in situ from carbonyl compounds, amines, and isonitriles.[30,31] This new reaction demonstrates that this new class of heterocyclic compounds can be prepared under relatively mild reaction conditions from readily available alkynes in a process in which gold(I) catalyzes the formation of alkenyl azides by nucleophilic attack onto the alkynes, as has been shown in the formation of carboxamides.[10] In addition, gold presumably provides the Brønsted acid required for the protodeauration and final formation of tetrazoles from the intermediate alkenyl azides under anhydrous, catalytic conditions. Further work aimed at developing new catalysts for the synthesis of tetrazoles from alkynes is in progress.
