# Supplementary material for: Gold-Catalyzed Synthesis of Tetrazoles from Alkynes by C=C Bond Cleavage
Source: Angew Chem Int Ed Engl. 2013 Nov 13;52(50):13468–71. doi: 10.1002/anie.201308076 (PMC4499258; doi:10.1002/anie.201308076)
Supplement: Supplementary file 1 [file anie0052-13468-sd1.pdf]

Angewandte Chemie
Eine Zeitschrift der Gesellschaft Deutscher Chemiker

Supporting Information

© Wiley-VCH 2013

69451 Weinheim, Germany

Gold-Catalyzed Synthesis of Tetrazoles from Alkynes by C–C Bond Cleavage**

*Morgane Gaydou and Antonio M. Echavarren**

anie_201308076_sm_miscellaneous_information.pdf

SUPPORTING INFORMATION

Table of Contents

General Methods S-2

Experimental Procedures S-4

NMR Spectra S-16

X-ray Crystal Structure Determinations S-40

General Methods

All reactions were carried out under Ar. Solvents were dried using a Solvent Purification System (SPS) or by standard procedures.[1] Gold(I) catalysts were either purchased from Sigma-Aldrich or synthesized according to literature procedures. All other reagents were used without further purification as received from the commercial sources.

Analytical thin layer chromatography was carried out using TLC aluminum sheets with 0.2 mm of silica gel (Merck GF234) using UV light as the visualizing agent. Flash column chromatography purifications were carried out using C_{18}-reversed phase silica gel (40-63 μm).

NMR spectra were recorded at 23 ºC on either a Bruker Avance 400 Ultrashield (400 MHz for ^{1}H, and 100 MHz for ^{13}C) or a Bruker Avance 500 Ultrashield (500 MHz for ^{1}H, and 125 MHz for ^{13}C) spectrometer.

Mass spectra were recorded on a Waters Micromass LCT Premier (ESI), Waters Micromass GCT (EI, CI) and Bruker Daltonics Autoflex (MALDI) spectrometers.

Melting points were determined using a Büchi melting point apparatus.

Crystal structure determinations were carried out using a Bruker-Nonius diffractomer equipped with an APPEX 2 4K CCD area detector, a FR591 rotating anode with MoK_a radiation, Montel mirrors as monochromator and a Kryoflex low temperature device (T = −173 ºC). Full-sphere data collection was used with w and j scans. *Programs used*: Data collection APEX-2, data reduction Bruker Saint V/.60A and absorption correction SADABS. Structure Solution and Refinement: Crystal structure solution was achieved using direct methods as implement in SHELXTL and

[1] Armarego, W. L. F.; Perrin, D. D. *Purification of Laboratory Chemicals*, Elsevier Science, Bath, **2003**.

visualized using the program XP. Missing atoms were subsequently located from difference Fourier synthesis and added to the atom list. Least-squares refinement on F2 using all measured intensities was carried out using the program SHELXTL. All non hydrogen atoms were refined including anisotropic displacement parameters.

Experimental Procedures.

General procedure for the formation of gold(I)-tetrazole complexes.

Cationic catalyst **A**, (acetonitrile)[(2-biphenyl)di-*tert*-butylphosphine]gold(I) hexafluoroantimonate, (0.1 mmol) and $TMSN_3$ (0.2 mmol) were suspended in CH_2Cl_2 (1 mL). The alkyne (0.1 mmol) was added and the reaction was stirred at room temperature (23 ªC) for 12 h. The solvent was evaporated and precipitation of the crude in a cyclohexane/EtOAc mixture gave the gold(I)-tetrazole complex as a white solid.

Complex 9a.

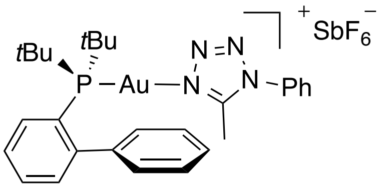

Alkyne = phenylacetylene. Yield 72%.

mp 224–226 ºC. ^{1}H NMR (500 MHz, CD_2Cl_2) δ 7.98–7.94 (m, 1H), 7.75–7.73 (m, 3H), 7.66–7.62 (m, 2H), 7.58–7.56 (m, 2H), 7.36–7.28 (m, 5H), 6.95–6.91 (m, 1H), 2.58 (s, 3H), 1.53 (s, 9H), 1.49 (s, 9H). ^{13}C NMR (126 MHz, CD_2Cl_2) δ 149.3 (s), 143.7 (s), 133.7 (d, J = 7.5 Hz), 132.4 (s), 132.0 (s), 131.1 (s), 130.1 (s), 129.1 (s), 128.1 (d, J = 8.0 Hz), 127.4 (s), 125.09 (s), 38.6 (d, J = 27.1 Hz), 31.2 (d, J = 5.7 Hz), 11.1 (s). ^{31}P NMR (203 MHz, CD_2Cl_2) δ 61.42. HRMS-ESI: *m*/*z*: calcd for $C_{28}H_{35}AuN_4P$ (M^+-SbF_6): 655.2272, found: 655.2260.

Complex 9b.

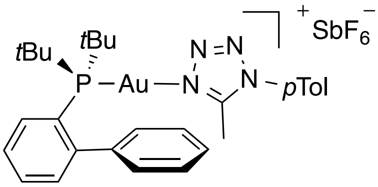

Alkyne = 4-ethynyltoluene. Yield 53%.

mp 213–215 ºC. ^{1}H NMR (500 MHz, CD_2Cl_2) δ 7.98–7.94 (m, 1H), 7.64–7.62 (m, 2H), 7.53–7.51 (m, 2H), 7.44–7.43 (m, 2H), 7.34–7.28 (m, 5H), 6.94–6.91 (m, 1H), 2.55 (s, 3H), 2.52 (s, 3H), 1.52 (s, 9H), 1.49 (s, 9H). ^{13}C NMR (126 MHz, CD_2Cl_2) δ 153.8 (d, J = 3.4 Hz), 149.4 (d, J = 12.0 Hz) 143.7 (d, J = 6.5 Hz), 143.4 (s), 133.9 (d,

J = 4.1 Hz), 133.7 (d, *J* = 7.4 Hz), 131.9 (d, *J* = 2.6 Hz), 131.5 (s), 130.1 (s), 129.1 (s), 128.9 (d, *J* = 4.0 Hz), 128.1 (d, *J* = 7.8 Hz), 127.4 (s), 124.9 (s), 38.6 (d, *J* = 27.1 Hz), 31.2 (dd, *J* = 12.2, 6.1 Hz), 21.5 (s), 11.1 (s). ^{31}P NMR (203 MHz, CD_2Cl_2) δ 61.34. HRMS-ESI: *m*/*z*: calcd for $C_{29}H_{37}AuN_4P$ (M^+-SbF_6): 669.2409, found: 669.2416.

Complex 9c.

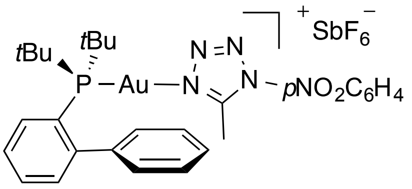

Alkyne = 1-ethynyl-4-nitrobenzene. Yield 45%.

mp 210–213. ^{1}H NMR (500 MHz, CD_2Cl_2) δ 8.57–8.55 (m, 2H), 7.97–7.94 (m, 1H), 7.87–7.86 (m, 2H), 7.66–7.61 (m, 2H), 7.38–7.35 (m, 3H), 7.31–7.29 (m, 2H), 6.99–6.96 (m, 1H), 2.67 (s, 3H), 1.52 (s, 9H), 1.49 (s, 9H). ^{13}C NMR (126 MHz, CD_2Cl_2) δ 154.3 (s), 149.9 (s), 149.4 (d, *J* = 12.0 Hz), 143.6 (d, *J* = 6.5 Hz), 137.2 (s), 133.8 (d, *J* = 4.1 Hz), 133.7 (d, *J* = 7.9 Hz), 131.9 (d, *J* = 2.5 Hz), 130.1 (s), 129.2 (s), 128.1 (d, *J* = 7.6 Hz), 127.6 (s), 126.3 (d, *J* = 3.7 Hz), 124.4 (s), 124.0 (s), 38.7 (s), 38.6 (d, *J* = 27.0 Hz), 31.2 (d, *J* = 6.2 Hz), 11.3 (s). ^{31}P NMR (203 MHz, CD_2Cl_2) δ 61.28. HRMS-ESI: *m*/*z*: calcd for $C_{28}H_{34}AuN_5O_2P$ (M^+-SbF_6): 700.2108, found: 700.2110.

Optimization Studies.

Solvent screening.

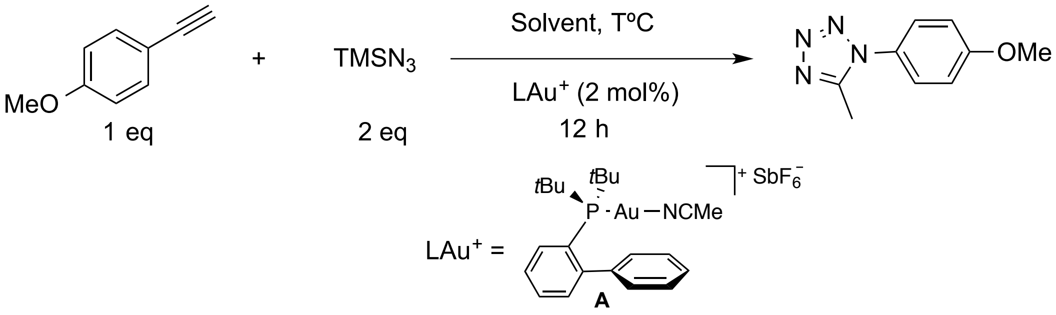

Entry	Solvent	T [ºC]	Yield [%][a]
1	MeCN	23	–

2	DCM	40	–
3	DCE	80	40
4	MeCN	80	8
5	DCE	80 (MW, 90 min)	36
6	DCE	110	38
7	Toluene	110	9

[a] Determined by ^{1}H NMR using diphenylmethane as internal standard. Conversions < 100%.

Catalyst screening.

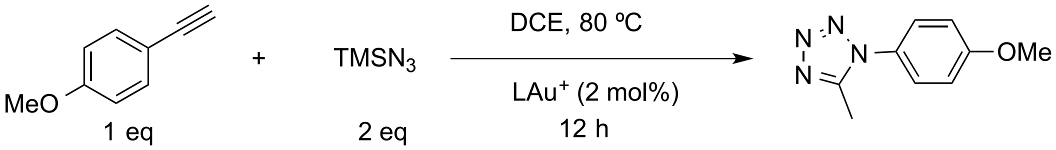

Entry	$[AuL]^+$	Yield [%]
1	**A**	40[a]
2	**B**	8[a]
3	**C**	7[a]
5	**D**	–[b]
6	**D'**	–[b]
4	**E**	–[b]
7	**F**	15[b]
8	**G**	18[b]
9	$AuCl(PPh_3)/Ag_2CO_3$	–[a]

[a] Determined by ^{1}H NMR using diphenylmethane as internal standard. [b] Determined by ^{1}H NMR using 1,4-diacetylbenzene as internal standard. Conversions < 100%.

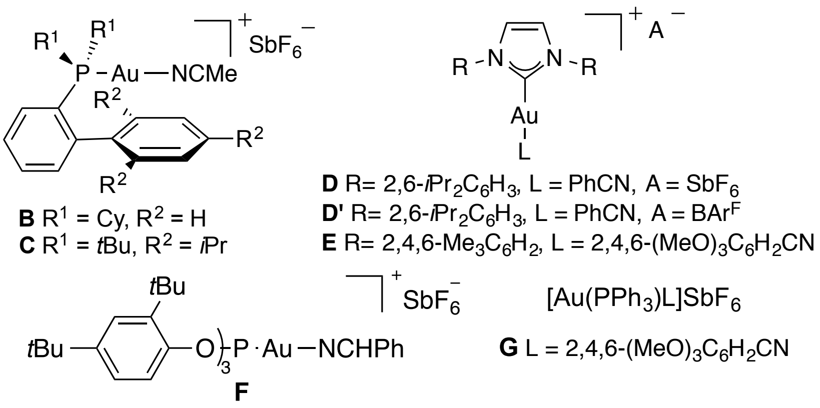

Catalyst loading.

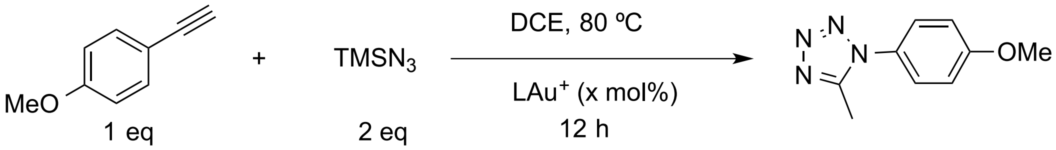

Entry	**A** (mol %)	Yield [%][a]
1	2	40[b]
2	5	48[b]
3	10	59

[a] Determined by ^{1}H NMR using diphenylmethane as internal standard. [b] Conversion < 100%..

Additive loading.

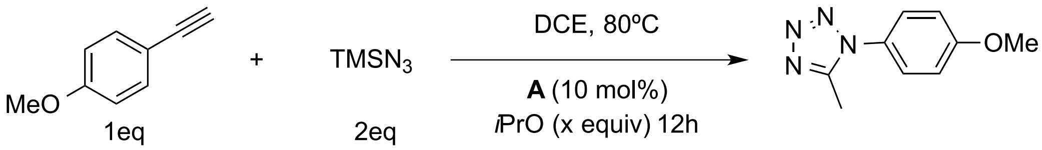

Entry	*i*PrOH (x eq)	Yield [%][a]
1	2	68
2	4	80
3	6	78
4	8	81
5	10	79

[a] Determined by ^{1}H NMR using diphenylmethane as internal standard.

Mechanistic Study.

Complex 9a-*d*.

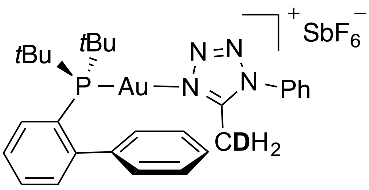

9a-*d*

The cationic gold(I) catalyst **A** (0.05 mmol) and $TMSN_3$ (0.1 mmol) were suspended in CH_2Cl_2 (0.5 mL). The phenylacetylene-*d* (0.05 mmol) was then added and the reaction was stirred at room temperature for 12 h. The solvent was evaporated and precipitation of the crude in a cyclohexane/EtOAc mixture gave complex **9a-*d*** (35%) as a white solid.

^{1}H NMR (500 MHz, $CDCl_3$) δ 7.93–7.90 (m, 1H), 7.72–7.67 (m, 3H), 7.61–7.59 (m, 5H), 7.38–7.35 (m, 2H), 7.33–7.31 (m, 1H), 7.30–7.28 (m, 1H), 6.96–6.93 (m, 1H), 2.64–2.62 (m, 2H), 1.52 (s, 9H), 1.49 (s, 9H). ^{13}C NMR (126 MHz, CD_2Cl_2) δ 154.1 (s), 149.4 (d, J = 12.7 Hz), 143.1 (s), 133.5 (d, J = 7.4 Hz), 133.4 (d, J = 3.8 Hz), 132.4 (s), 131.9 (s), 131.6 (s), 130.7 (s), 129.8 (s), 129.0 (s), 127.6 (d, J = 7.6 Hz), 127.4 (s), 125.0 (s), 38.5 (d, J = 27.0 Hz), 31.1 (dd, J = 21.5, 6.5 Hz), 10.8 (t, J = 20.2 Hz). ^{31}P NMR (162 MHz, CD_2Cl_2) δ 61.14. HRMS-ESI: *m*/*z*: calcd for $C_{28}H_{34}AuDN_4P$ (M^+-SbF_6): 656.2321, found: 656.2322.

Complex 9a-*d*₂.

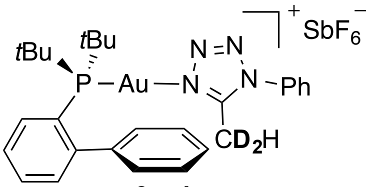

9a-d_2

The cationic gold(I) catalyst **A** (0.05 mmol) and $TMSN_3$ (0.1 mmol) were suspended in CH_2Cl_2 (0.5 mL). The phenylacetylene (0,05 mmol) and D_2O (0.05 mmol) were then added and the reaction was stirred at room temperature for 12 h. The solvent was evaporated and precipitation of the crude in a cyclohexane/EtOAc mixture gave complex **9a-*d*** (42%) as a white solid

^{1}H NMR (400 MHz, CD_2Cl_2) δ 7.96−7.93 (m, 1H), 7.90−7.86 (m, 1H), 7.63−7.57 (m, 4H), 7.55−7.50 (m, 4H), 7.33−7.30 (m, 2H), 7.21−7.19 (m, 2H), 2.59−2.54 (m, 1H), 1.41(s, 9H), 1.37 (s, 9H). ^{13}C NMR (101 MHz, CD_2Cl_2) δ 149.5 (d, J = 12.2 Hz), 142.7 (s), 133.7 (m), 131.8 (d, J = 2.4 Hz), 131.0 (s), 130.0 (s), 128.9 (t, J = 4.1 Hz), 128.6 (s), 127.9 (d, J = 7.4 Hz), 125.1 (s), 124.5 (s), 124.1 (s), 38.3 (d, J = 26.7 Hz), 31.1 (dd, J = 15.3, 6.6 Hz). ^{31}P NMR (162 MHz, CD_2Cl_2) δ 62.61. HRMS-ESI: *m/z*: calcd for $C_{20}H_{27}AuP$: 495.1501, found: 495.1510. We did not detected $(M\text{-SbF6})^{+}$: the additional loss of the tetrazole ligand was observed.

Reaction with acetophenone as the substrate

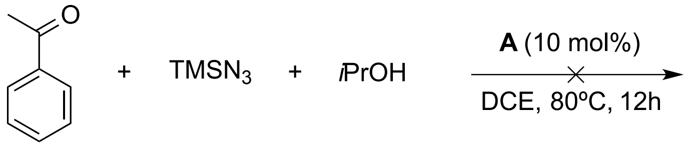

The cationic gold(I) catalyst **A** (0.02 mmol) and $TMSN_3$ (0.4 mmol) were suspended in DCE (1 mL). Then, acetophenone (0.2 mmol) in DCE (1 mL) and *i*PrOH (0.8 mmol) were added and the reaction was stirred at 80 ºC for 12 h. After cooling the reaction mixture at room temperature and the addition of a few drops of NEt_3, the solvent was evaporated. No conversion to ttetrazole **8a** and recovery of the acetophenone were observed, which demonstrates that acetophenones are not intermediates in this process.

Reactions with (1-azidovinyl)benzene (5a) as the substrate

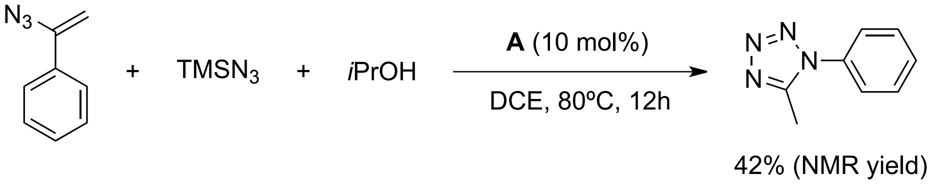

The cationic gold(I) catalyst **A** (0.02 mmol) and $TMSN_3$ (0.4 mmol) were suspended in DCE (1 mL). 1-azido-1-phenylethylene[2] (0.2 mmol) in DCE (1 mL) and *i*PrOH (0.8 mmol) were then added and the reaction was stirred at 80 ºC for 12 h. After cooling the reaction mixture at room temperature and the addition of a few drops of NEt_3, the solvent was evaporated yielding 5-methyl-1-phenyl-1*H*-tetrazole **8a** (42%, determined by ^{1}H NMR using diphenylmethane as internal standard). This result

[2] F. Shi, J. P. Waldo, Y. Chen, R. C. Larock, *Org. Lett.* **2008**, *10*, 2409–2412.

supports the proposed mechanism and allows to conclude that vinyl azides are intermediates in the gold(I)-catalyzed synthesis of tetrazoles.

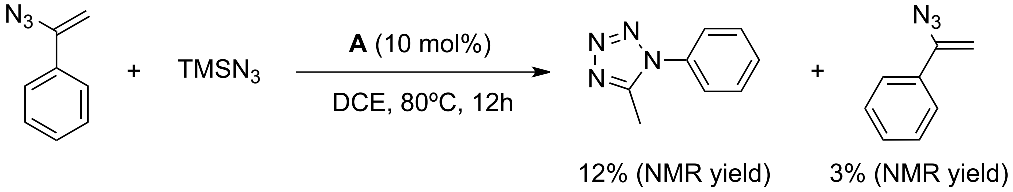

Using the procedure previously described but without the addition of *i*PrOH, 5-methyl-1-phenyl-1*H*-tetrazole **8a** was obtained in only 12 % yield (determined by ^{1}H NMR using diphenylmethane as internal standard).

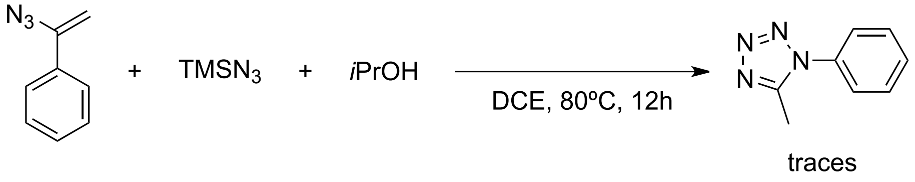

Using the procedure previously described but without the addition of the cationic gold(I) catalyst **A** in the reaction mixture, only traces of 5-methyl-1-phenyl-1*H*-tetrazole **8a** were observed.

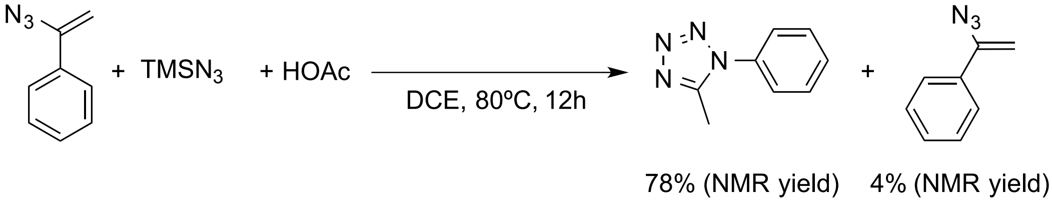

Using the procedure previously described but without the addition of the cationic gold(I) catalyst **A** and the addition of 2 equiv of HOAc, 5-methyl-1-phenyl-1*H*-tetrazole **8a** (78%, determined by ^{1}H NMR using diphenylmethane as internal standard) was obtained.

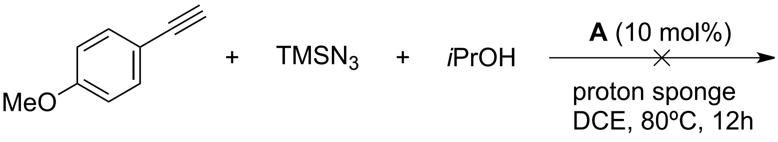

A control experiment was performed by adding proton sponge (2 equiv) as the base to the reaction mixture containing 4-ethynylanisol (1 equiv), $TMSN_3$ (2 equiv), *i*PrOH (4 equiv), and gold(I) catalyst **A** (10 mol%.) in DCE (0.1 M, 80 ºC, 12h). Under these conditions, no reaction was observed.

General Procedure for the preparation of 1,5-disubstituted tetrazoles 8.

The cationic gold(I) catalyst **A** (0.02 mmol) and $TMSN_3$ (0.4 mmol) were suspended in DCE (1 mL). The alkyne (0.2 mmol) in DCE (1 mL) and *i*PrOH (0.8 mmol) were then added and the reaction was stirred at 80 ºC for 12 h. After cooling the reaction mixture at room temperature and the addition of a few drops of NEt_3, the solvent was evaporated and the crude was purified using C_{18}-reversed phase silica gel ($MeCN/H_2O$, 1/2 to 1/1) yielding the 1,5-disubstituted tetrazole.

5-Methyl-1-phenyl-1*H*-tetrazole (8a).[3]

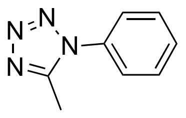

White solid. Yield 49%.

mp 102–104 ºC. ^{1}H NMR (400 MHz, $CDCl_3$) δ 7.62–7.57 (m, 3H), 7.48–7.46 (m, 2H), 2.62 (s, 3H). ^{13}C NMR (100 MHz, $CDCl_3$) δ 151.7, 134.0, 130.5, 130.1, 124.7, 10.0. HRMS-ESI: *m*/*z*: calcd for $C_8H_8N_4Na$ (M^++Na): 183.0644, found: 183.0641.

5-Methyl-1-(*p*-tolyl)-1*H*-tetrazole (8b).[3]

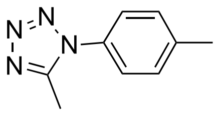

Yellowish solid. Yield 41%.

mp 110–113 ºC. ^{1}H NMR (400 MHz, $CDCl_3$) δ 7.39–7.37 (m, 2H), 7.34–7.33 (m, 2H), 2.60 (s, 3H), 2.47 (s, 3H). ^{13}C NMR (100 MHz, $CDCl_3$) δ 151.7, 140.9, 131.5, 130.6, 124.6, 21.4, 9.9. HRMS-ESI: *m*/*z*: calcd for $C_9H_{10}N_4Na$ (M^++Na): 197.0805, found: 197.0798.

1-(1-Azidovinyl)-4-nitrobenzene (5c).[4]

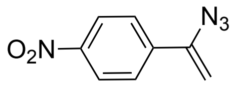

Yellow oil. Yield 23%.

^{1}H NMR (400 MHz, $CDCl_3$) δ 8.21–8.19 (m, 2H), 7.74–7.72 (m, 2H), 5.64 (d, *J* = 3.0 Hz, 1H), 5.15 (d, *J* = 3.0 Hz, 1H). ^{13}C NMR (100 MHz, $CDCl_3$) δ 148.2, 143.5, 140.3, 126.5, 123.9, 101.2. The ^{1}H NMR data are identical to that reported in ref. 4b.

1-(4-Methoxyphenyl)-5-methyl-1*H*-tetrazole (8d).[3]

---

[3] A.-A. S. El-Ahl, F. A. Amer, A. H. Elbeheery, *Phosphorus, Sulfur, and Silicon and Related Elements*, **2011**, *186*, 2226–2235.
[4] (a) A. Hassner, D. J. Anderson, R. H. Reuss, *Tetrahedron Lett.* **1977**, 2463–2466; (b) D. Brown, G. A. Brown, M. Andrews, J- M. Large, D. Urban, C. P. Butts, N. J. Hales, T. Gallagher, *J. Chem. Soc., Perkin Trans. 1* **2002**, 2014–2021.

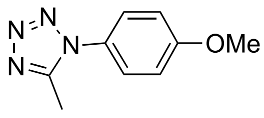

White solid. Yield 70%.

mp 96–98 ºC. ^{1}H NMR (400 MHz, $CDCl_3$) δ 7.37–7.35 (m, 2H), 7.07–7.06 (m, 2H), 3.89 (s, 3H), 2.57 (s, 3H). ^{13}C NMR (100 MHz, CD_2Cl_2) δ 161.0, 151.8, 126.7, 126.3, 115.2, 55.9, 9.8. HRMS-ESI: *m*/*z*: calcd for $C_9H_{10}N_4NaO$ (M^++Na): 213.0749, found: 213.0747.

1-(3-Methoxyphenyl)-5-methyl-1*H*-tetrazole (8e).

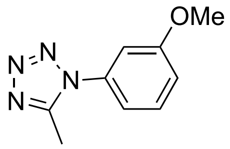

Yellow oil. Yield 36%.

^{1}H NMR (400 MHz, $CDCl_3$) δ 7.50–7.46 (m, 1H), 7.11–7.09 (m, 1H), 7.03–7.00 (m, 2H), 3.87 (s, 3H), 2.62 (s, 3H). ^{13}C NMR (100 MHz, $CDCl_3$) δ 160.6, 151.5, 134.8, 130.7, 116.4, 116.0, 110.5, 55.7, 9.9. HRMS-ESI: *m*/*z*: calcd for $C_9H_{10}N_4NaO$ (M^++Na): 213.0748, found: 213.0747.

1-(2-Methoxyphenyl)-5-methyl-1*H*-tetrazole (8f).

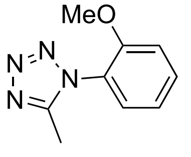

Yellow oil. Yield 38%.

^{1}H NMR (400 MHz, $CDCl_3$) δ 7.57–7.54 (m, 1H), 7.37–7.35 (m, 1H), 7.15–7.10 (m, 2H), 3.83 (s, 3H), 2.45 (s, 3H). ^{13}C NMR (100 MHz, $CDCl_3$) δ 153.9, 153.5, 132.5, 128.2, 122.6, 121.3, 112.5, 56.1, 9.2. HRMS-ESI: *m*/*z*: calcd for $C_9H_{10}N_4NaO$ (M^++Na): 213.0755, found: 213.0747.

1-(4-Bromophenyl)-5-methyl-1*H*-tetrazole (8g).[3]

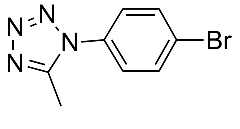

Yellow solid. Yield 36%.

mp 125–126 ºC. ^{1}H NMR (400 MHz, $CDCl_3$) δ 7.75–7.73 (m, 2H), 7.38–7.36 (m, 2H), 2.62 (s, 3H). ^{13}C NMR (100 MHz, $CDCl_3$) δ 151.6, 133.4, 133.0, 126.1, 124.7, 10.0. HRMS-ESI: *m*/*z*: calcd for $C_8H_8BrN_4$ (M^++H): 238.9934, found: 238.9927.

1-(4-(*tert*-Butyl)phenyl)-5-methyl-1*H*-tetrazole (8h).

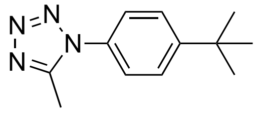

Yellow oil. Yield 54%.

^{1}H NMR (400 MHz, $CDCl_3$) δ 7.59–7.58 (m, 2H), 7.58–7.37 (m, 2H), 2.61 (s, 3H), 1.38 (s, 9H). ^{13}C NMR (100 MHz, $CDCl_3$) δ 154.1, 151.8, 131.5, 127.1, 124.4, 35.2, 31.4, 10.0. HRMS-ESI: *m*/*z*: calcd for $C_{12}H_{16}N_4Na$ (M^++Na): 239.1266, found: 239.1267.

1-([1,1'-Biphenyl]-4-yl)-5-methyl-1*H*-tetrazole (8i).

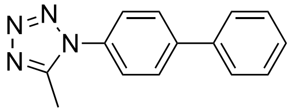

White solid. Yield 56%.

mp 177–180 ºC. ^{1}H NMR (400 MHz, $CDCl_3$) δ 7.82–7.80 (m, 2H), 7.66–7.63 (m, 2H), 7.57–7.42 (m, 5H), 2.68 (s, 3H). ^{13}C NMR (100 MHz, $CDCl_3$) δ 151.8, 143.7, 139.5, 133.1, 129.3, 128.8, 127.5, 125.1, 10.1. HRMS-ESI: *m*/*z*: calcd for $C_{14}H_{12}N_4Na$ (M^++Na): 259.0951, found: 259.0954.

5-Methyl-1-(naphthalen-1-yl)-1*H*-tetrazole (8j).[3]

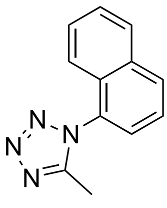

Yellow solid. Yield 49%.

mp 101–104 ºC. ^{1}H NMR (400 MHz, $CDCl_3$) δ 8.13–8.11 (m, 1H), 8.02–8.01 (m, 1H), 7.66–7.61 (m, 2H), 7.58–7.55, (m, 1H), 7.51–7.49 (m, 1H), 7.17–7.15 (m, 1H), 2.44 (s, 3H). ^{13}C NMR (100 MHz, $CDCl_3$) δ 153.4, 134.2, 131.6, 129.0, 128.5, 127.5, 125.0, 121.5, 9.1. HRMS-ESI: *m*/*z*: calcd for $C_{12}H_{10}N_4Na$ (M^++Na): 233.0798, found: 233.0798.

5-Methyl-1-(4-(trifluoromethyl)phenyl)-1*H*-tetrazole (8k).

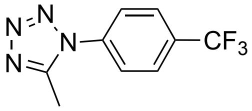

Yellow oil. Yield 18%.

^{1}H NMR (400 MHz, $CDCl_3$) δ 7.90–7.88 (m, 2H), 7.67–7.66 (m, 2H), 2.68 (s, 3H). ^{13}C NMR (100 MHz, $CDCl_3$) δ 151.6, 136.8, 129.3, 127.4, 126.5, 124.9, 10.1. HRMS-ESI: *m*/*z*: calcd for $C_9H_8F_3N_4$ (M^++H): 229.0698, found: 229.0696.

5-Methyl-1-(thiophen-3-yl)-1*H*-tetrazole (8l).

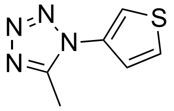

Yellow oil. Yield 51%.

^{1}H NMR (400 MHz, $CDCl_3$) δ 7.59–7.57 (m, 2H), 7.35–7.34 (m, 1H), 2.69 (s, 3H). ^{13}C NMR (100 MHz, $CDCl_3$) δ 151.4, 131.9, 127.8, 123.1, 119.7, 10.0. HRMS-ESI: *m*/*z*: calcd for $C_6H_6N_4NaS$ (M^++Na): 189.0204, found: 189.0205.

1-Cyclohexyl-5-methyl-1*H*-tetrazole (8m).[5]

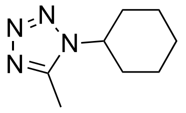

White solid. Yield 76%.

mp 130−131 ºC. ^{1}H NMR (400 MHz, $CDCl_3$) δ 4.16−4.09 (m, 1H), 2.55 (s, 3H) 2.04−1.94 (m, 6H), 1.79−1.75 (m, 1H), 1.45−1.39 (m, 3H). ^{13}C NMR (100 MHz, $CDCl_3$) δ 150.6, 57.9, 32.7, 25.4, 25.0, 9.2. HRMS-ESI: *m*/*z*: calcd for $C_8H_{14}N_4Na$ (M^++Na): 189.1117, found: 189.1111.

5-Methyl-1-propyl-1*H*-tetrazole and 1-methyl-5-propyl-1*H*-tetrazole (8n, 8n').[6]

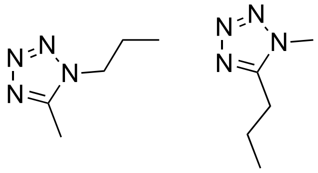

10: 1

Yellow oil, inseparable mixture of isomers. Yield 50% (10: 1).

5-methyl-1-propyl-1*H*-tetrazole: ^{1}H NMR (400 MHz, $CDCl_3$) δ 4.25 (t, *J* = 9.5 Hz, 2H), 2.58 (s, 3H), 1.97 (h, *J* = 7.4 Hz, 2H), 1.00 (t, *J* = 7.5 Hz, 3H). ^{13}C NMR (100 MHz, $CDCl_3$) δ 151.3, 48.7, 23.0, 11.0, 8.9.

1-methyl-5-propyl-1*H*-tetrazole: ^{1}H NMR (400 MHz, $CDCl_3$) δ 4.02 (s, 3H), 2.85 (t, *J* = 11 Hz, 2H), 1.88 (h, *J* = 7.4 Hz, 2H), 1.06 (t, *J* = 7.4 Hz, 3H). ^{13}C NMR (100 MHz, $CDCl_3$) δ 153.7, 47.0, 24.9, 13.7, 8.7.

HRMS-ESI: *m*/*z*: calcd for $C_5H_{11}N_4$ (M^++H): 127.0975, found: 127.0978.

1-Cyclopropyl-5-methyl-1*H*-tetrazole and 5-cyclopropyl-1-methyl-1*H*-tetrazole (8o, 8o').[7]

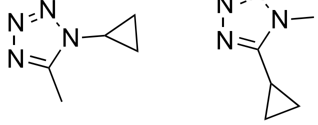

1: 3

Yellow oil, inseparable mixture of isomers. Yield 49% (1: 3).

1-cyclopropyl-5-methyl-1*H*-tetrazole: ^{1}H NMR (400 MHz, $CDCl_3$) δ 3.45 (tt, *J* = 6.2, 4.1 Hz, 1H), 2.60 (s, 3H), 1.27−1.24 (m, 4H). ^{13}C NMR (100 MHz, $CDCl_3$) δ 153.2, 47.1, 9.1, 8.8, 8.7.

5-cyclopropyl-1-methyl-1*H*-tetrazole: ^{1}H NMR (400 MHz, $CDCl_3$) d 4.06 (s, 3H), 1.86 (tt, *J* = 7.6, 6.0 Hz, 1H), 1.21−1.19 (m, 4H). ^{13}C NMR (100 MHz, $CDCl_3$) δ 157.2, 33.1, 28.0, 6.8, 3.8.

[5] E. K. Harvill, R. M. Herbst, E. C. Schreiner, C. W. Roberts, *J. Org. Chem.* **1950**, *15*, 662–670.
[6] K. Nishiyama, A. Watanabe, *Chem. Lett.* **1984**,455–458.
[7] L. E. Fikes, H. Shechter, *J. Org. Chem.* **1978**, *44*, 741–744.

HRMS-ESI: *m*/*z*: calcd for $C_5H_9N_4$ (M^++H): 125.0828, found: 125.0822.

NMR spectra.

Complex 9a.

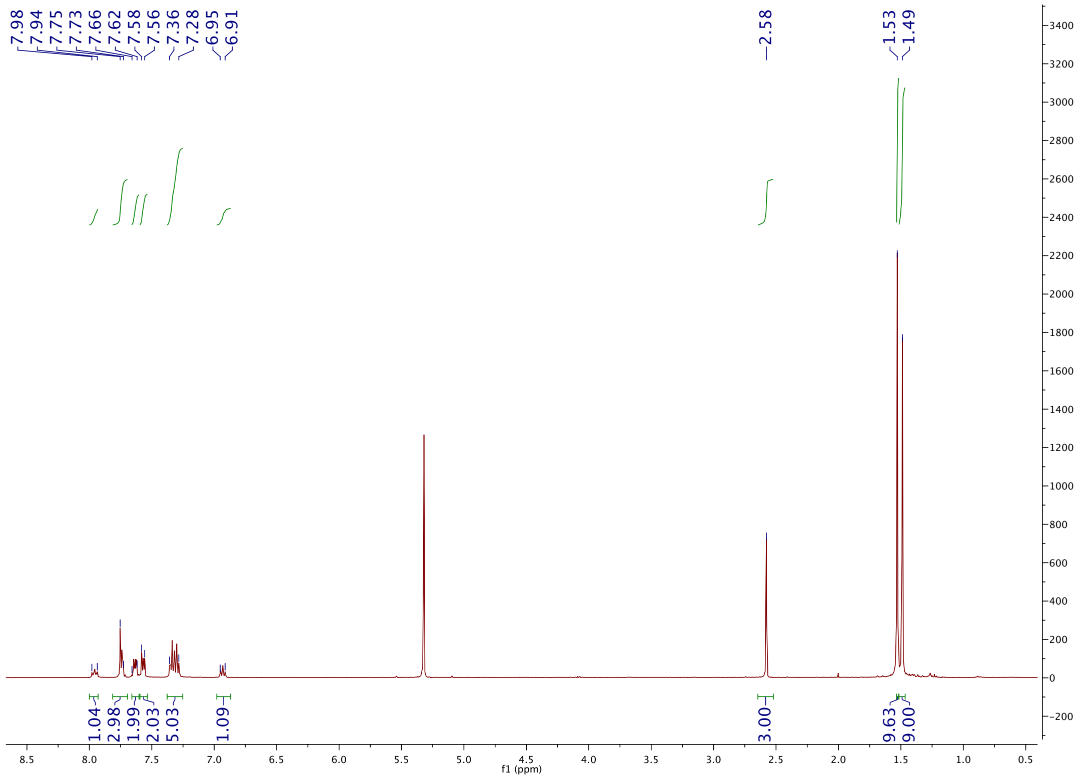

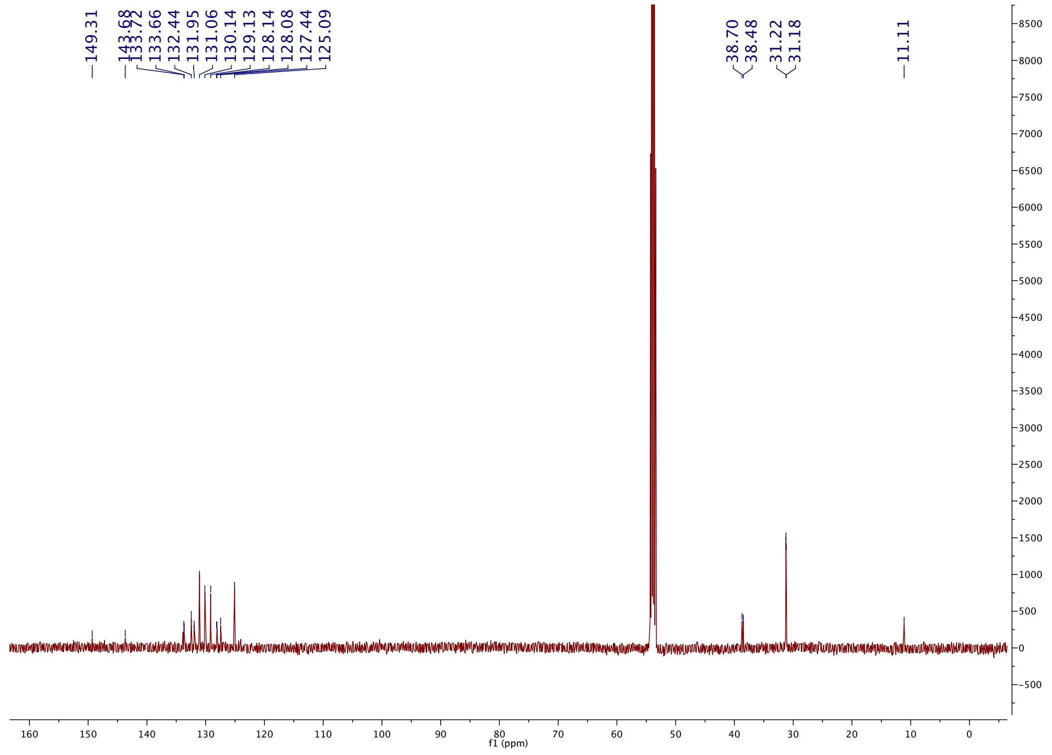

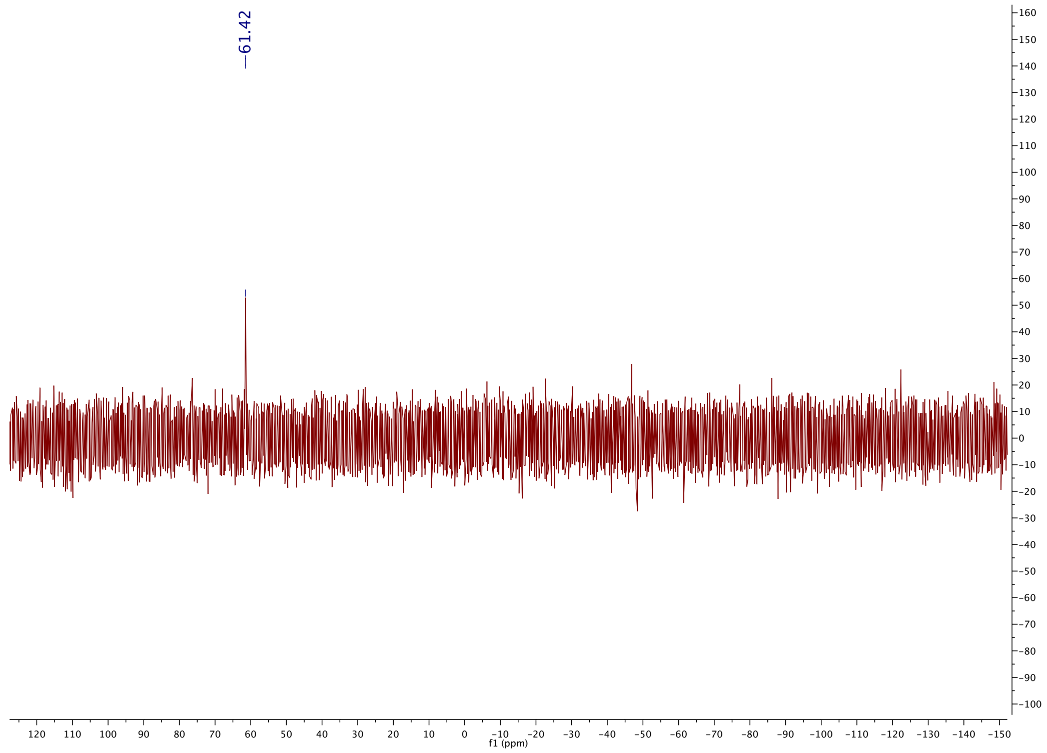

Complex 9b.

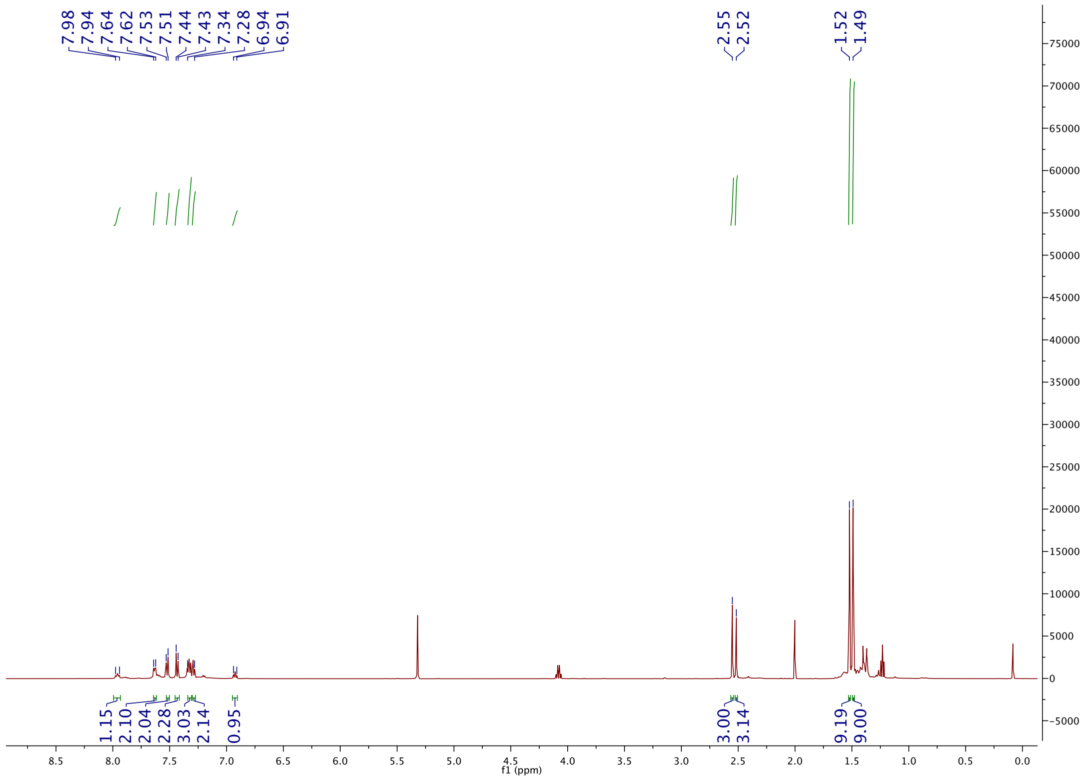

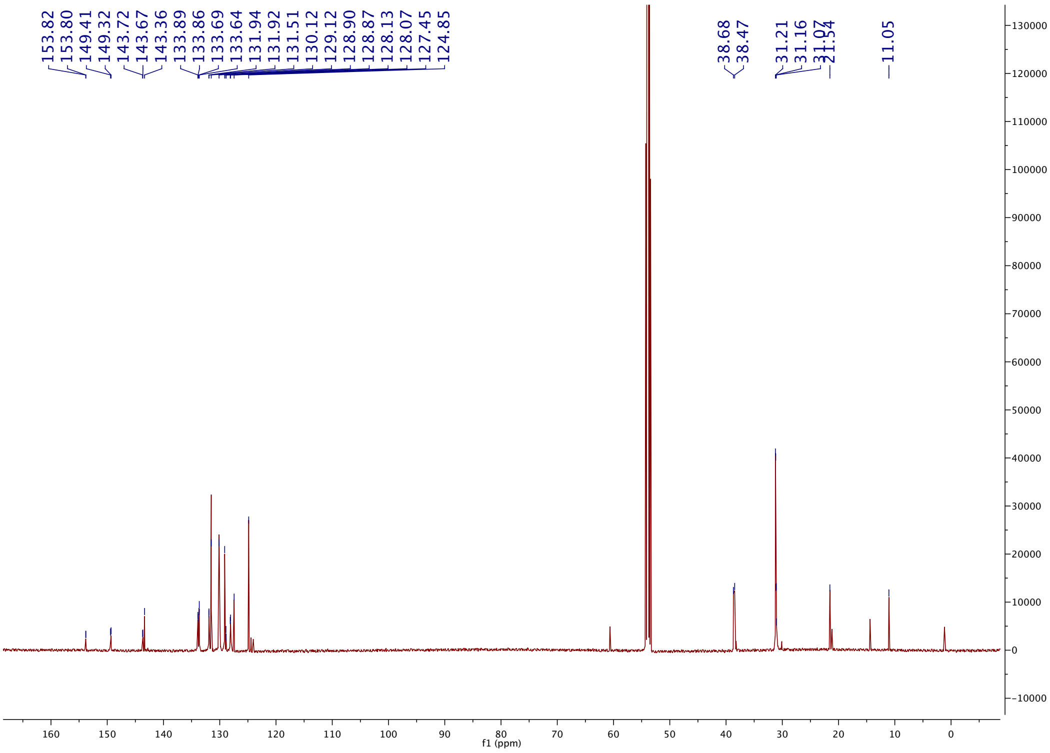
153.82
153.80
149.41
149.32
143.72
143.67
143.36
133.89
133.86
133.69
133.64
131.94
131.92
131.51
130.12
129.12
128.90
128.87
128.13
128.07
127.45
124.85
38.68
38.47
31.21
31.16
31.07
21.54
11.05
f1 (ppm)

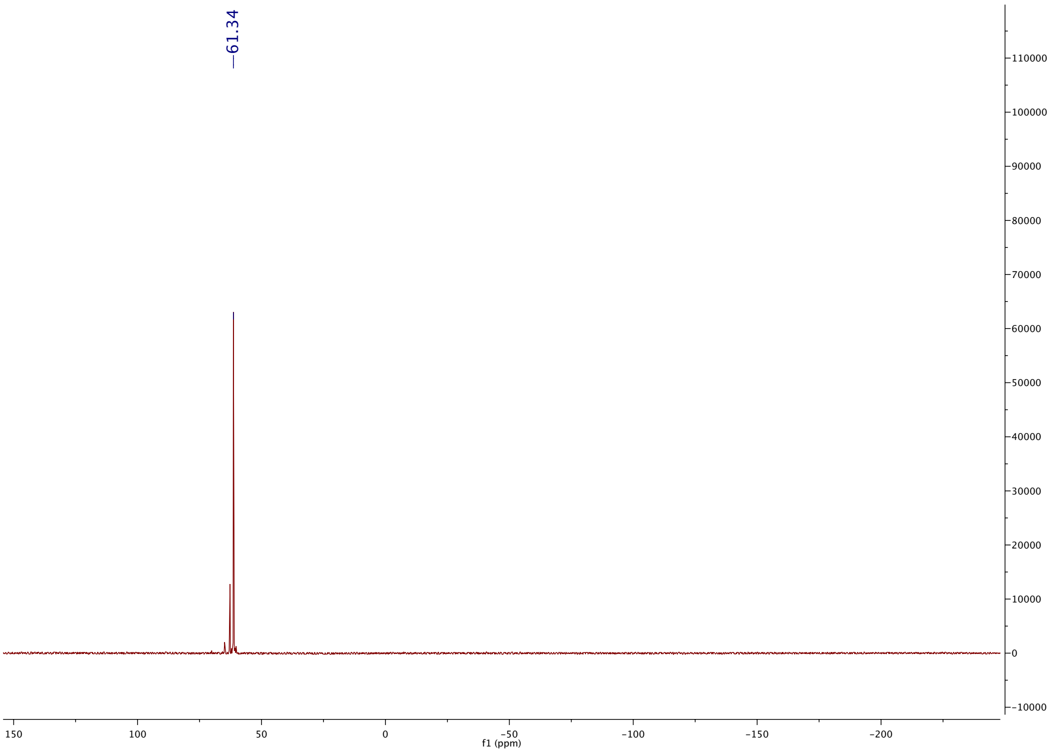
-61.34
f1 (ppm)

Complex 9c.

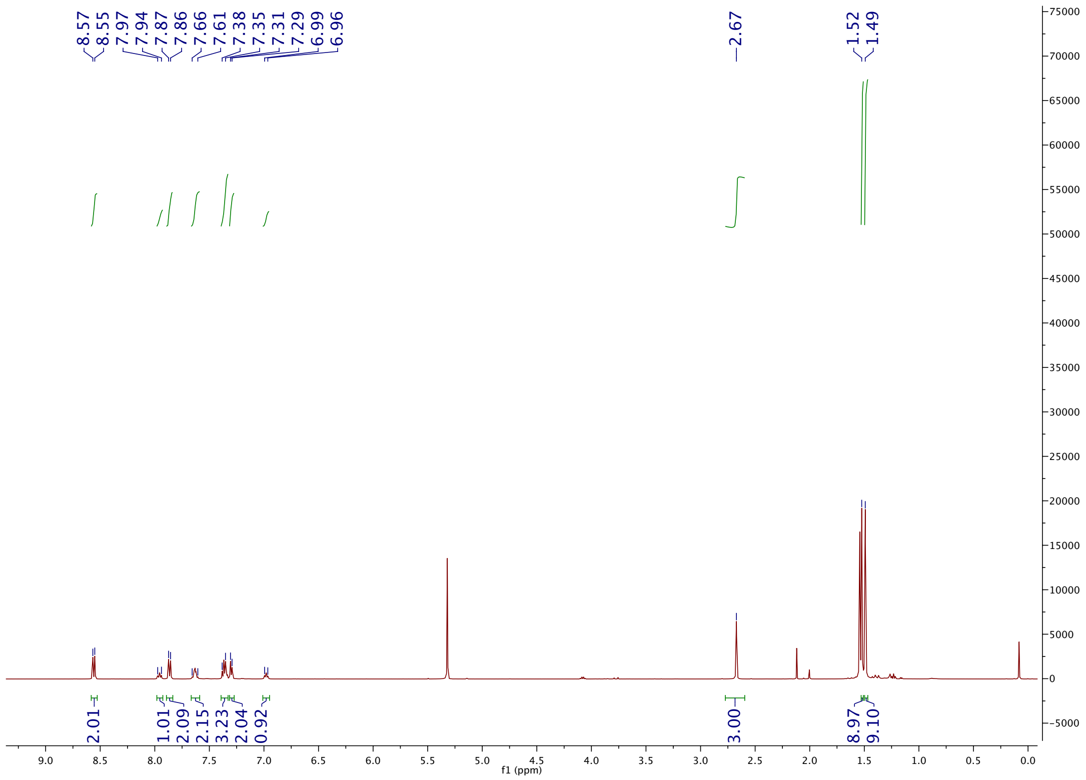
8.57
8.55
7.97
7.94
7.87
7.86
7.66
7.61
7.38
7.35
7.31
7.29
6.99
6.96
2.67
1.52
1.49
2.01
1.01
2.09
2.15
3.23
2.04
0.92
3.00
8.97
9.10
f1 (ppm)

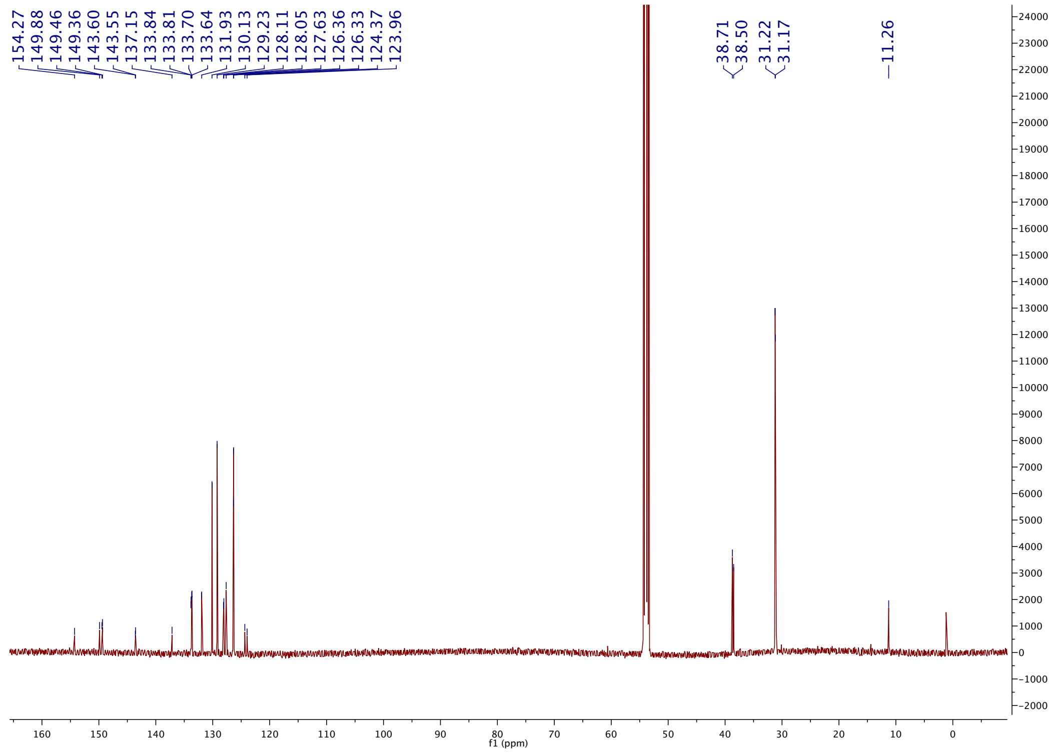
154.27
149.88
149.46
149.36
143.60
143.55
137.15
133.84
133.81
133.70
133.64
131.93
130.13
129.23
128.11
128.05
127.63
126.36
126.33
124.37
123.96
38.71
38.50
31.22
31.17
11.26
f1 (ppm)

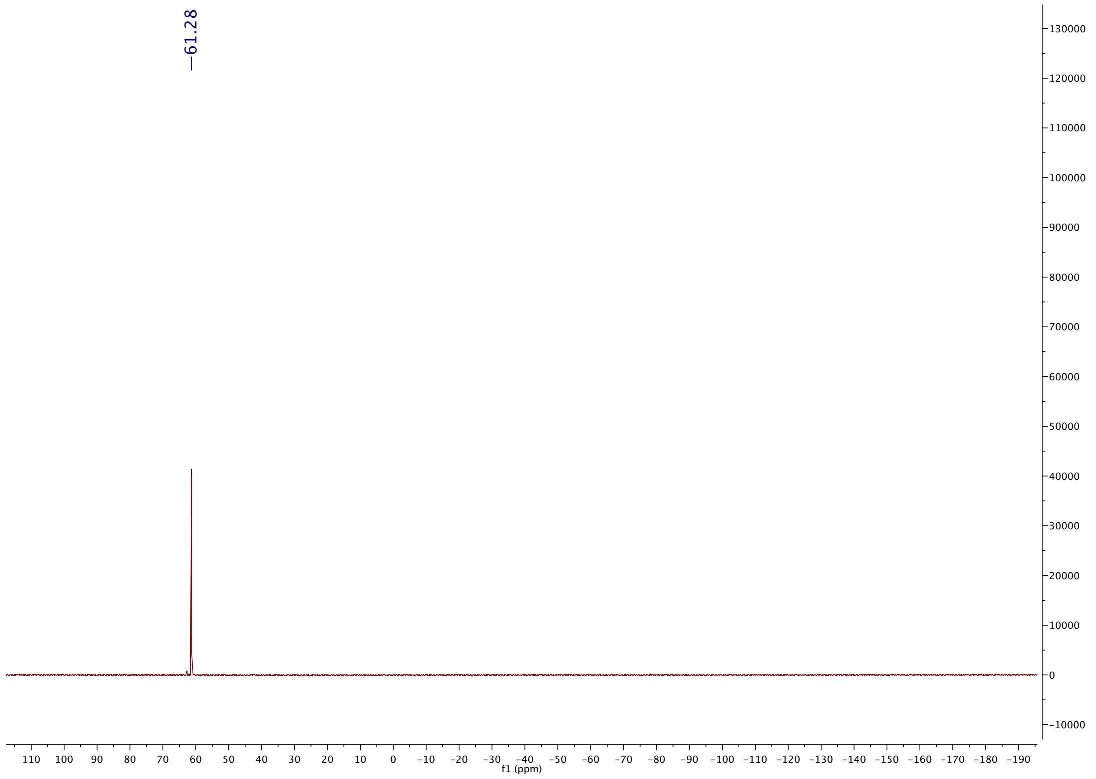

Mechanistic Study.

Complex 9a-*d*.

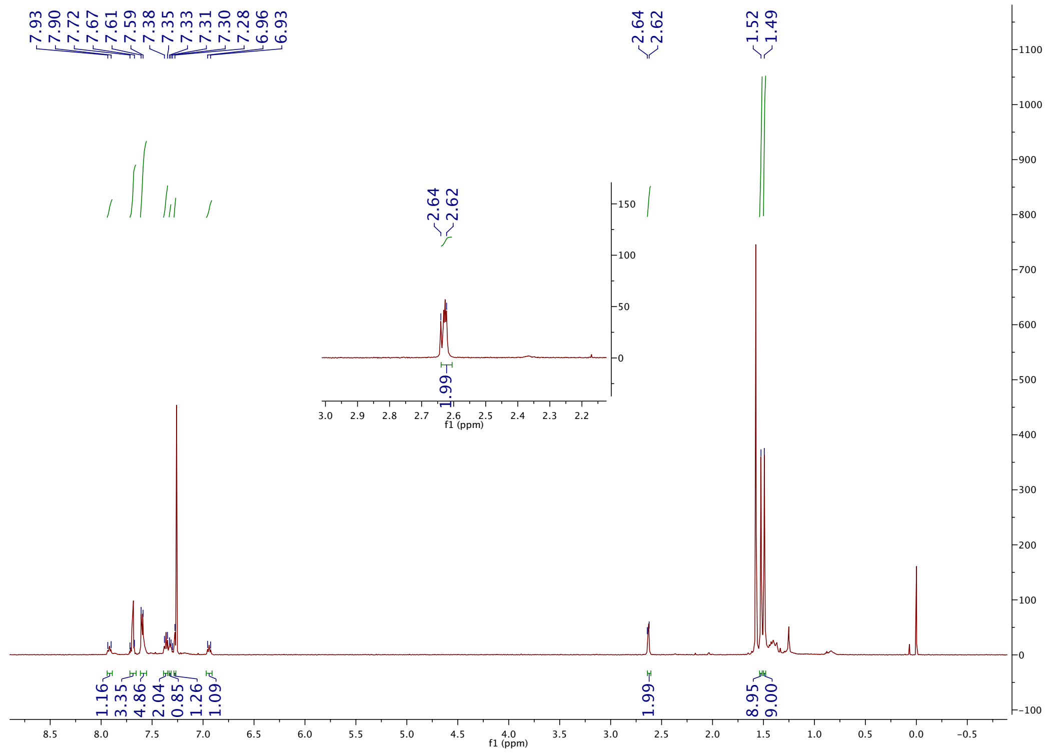

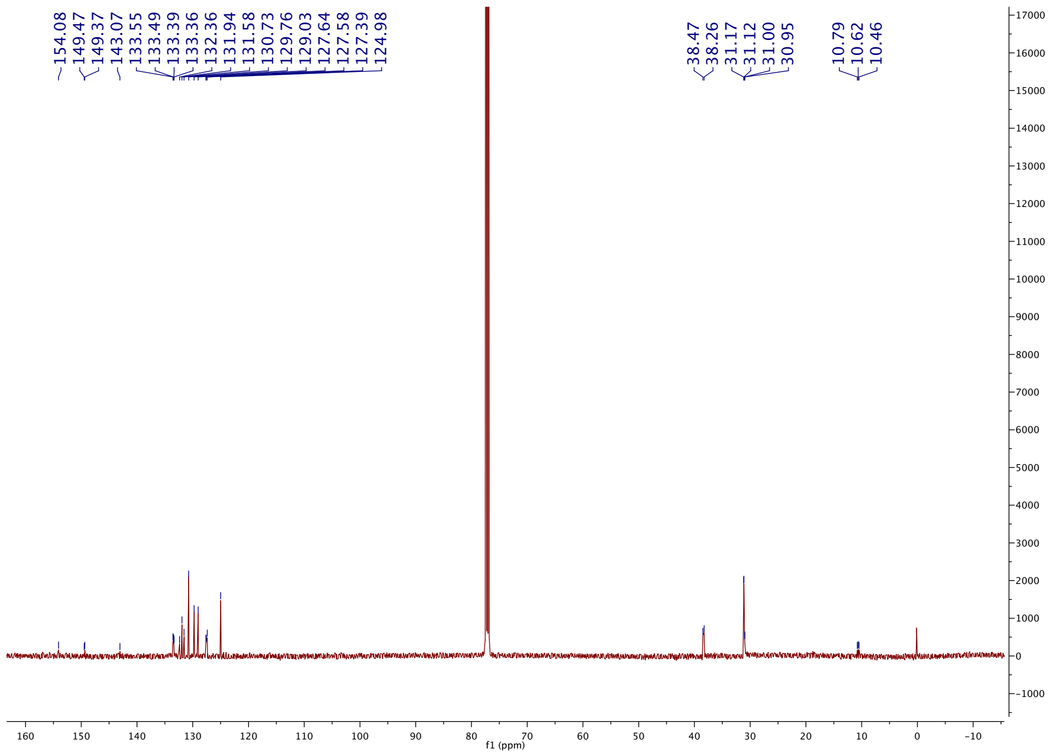
154.08
149.47
149.37
143.07
133.55
133.49
133.39
133.36
132.36
131.94
131.58
130.73
129.76
129.03
127.64
127.58
127.39
124.98
38.47
38.26
31.17
31.12
31.00
30.95
10.79
10.62
10.46
f1 (ppm)

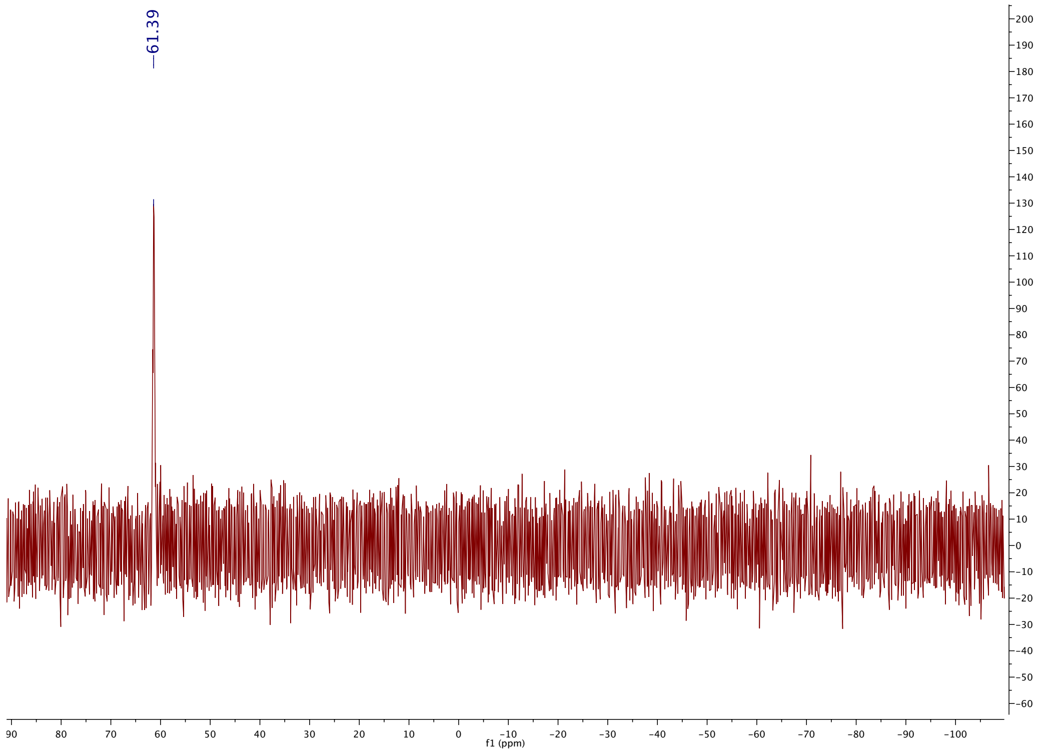
-61.39
f1 (ppm)

^{1}H NMR of Complex **9a-*d*** compared with that of **9a**.

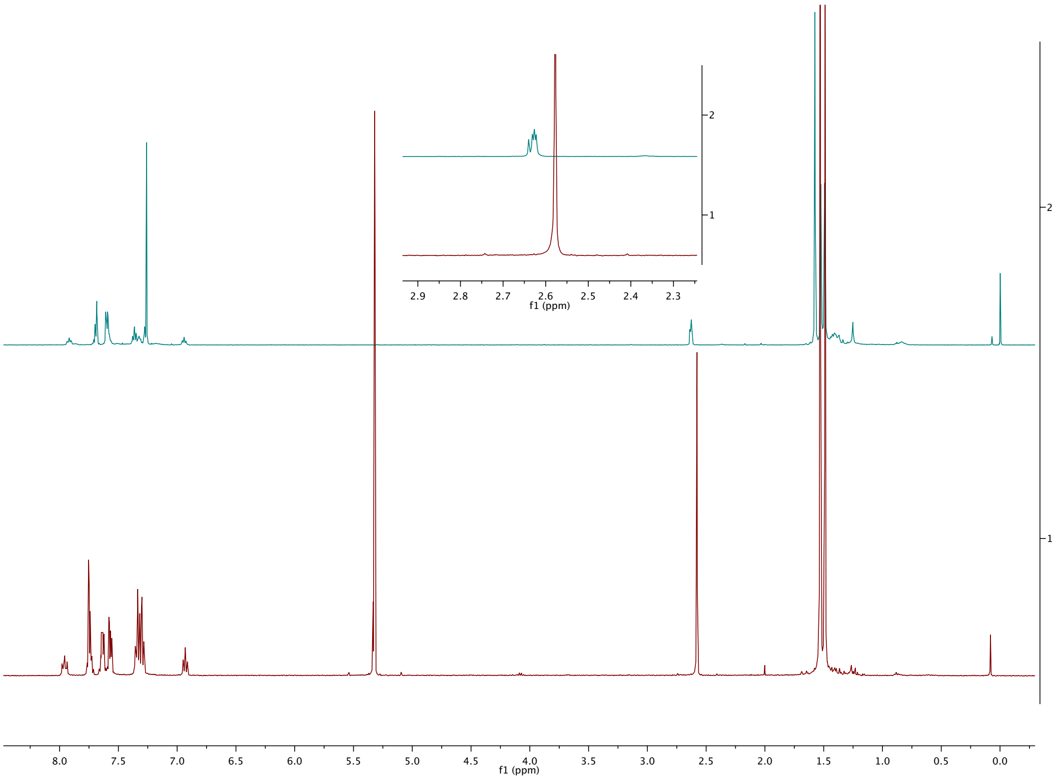

Complex 9a-*d*$_2$.

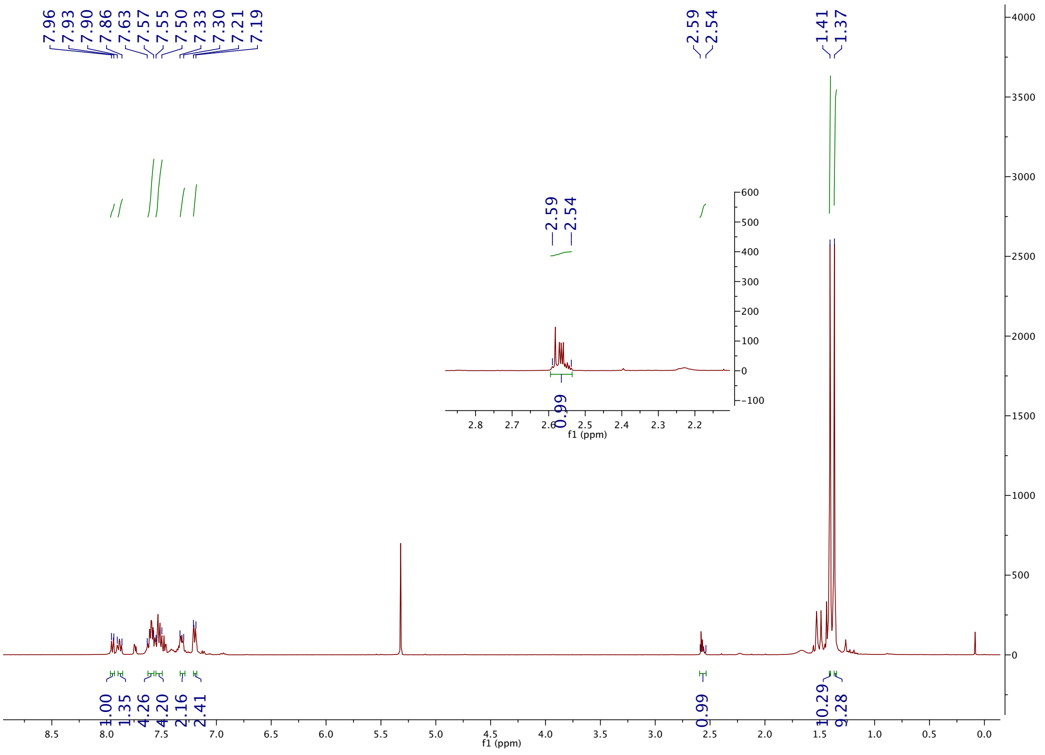

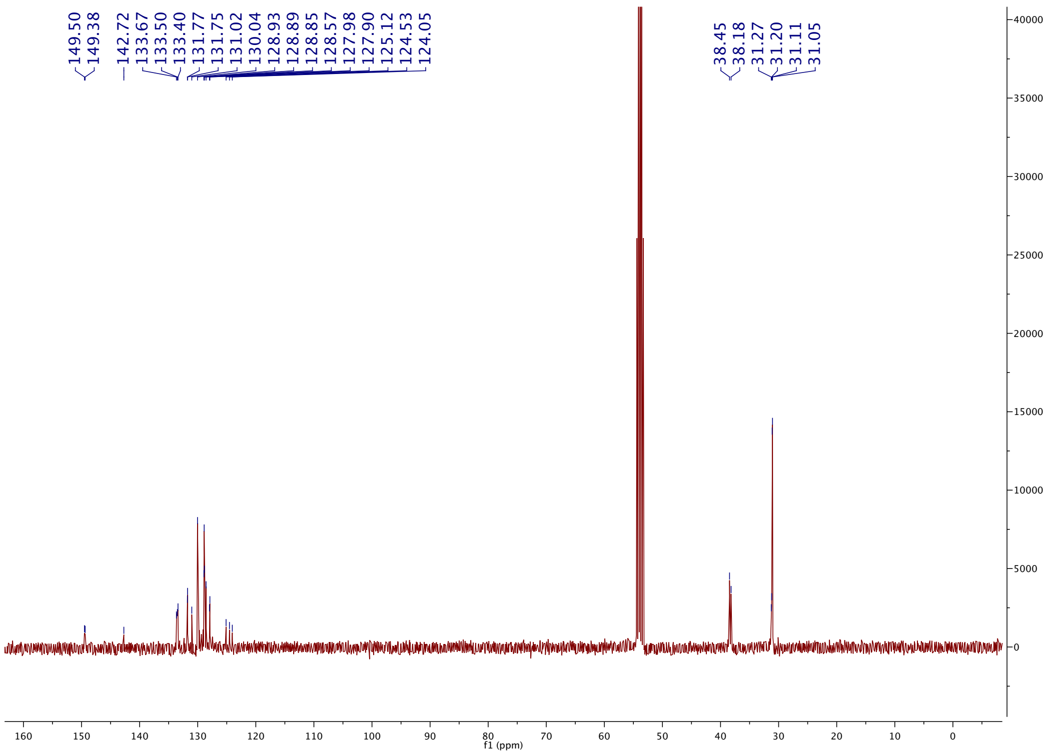
149.50
149.38
142.72
133.67
133.50
133.40
131.77
131.75
131.02
130.04
128.93
128.89
128.85
128.57
127.98
127.90
125.12
124.53
124.05
38.45
38.18
31.27
31.20
31.11
31.05
f1 (ppm)

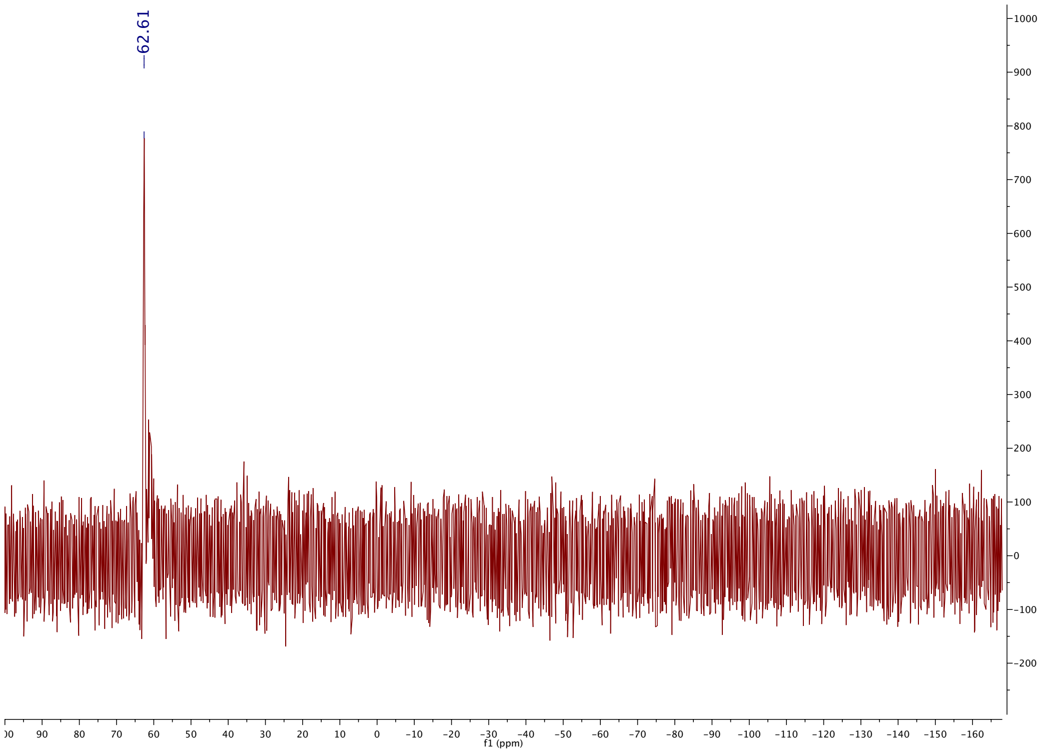
62.61
f1 (ppm)

^{1}H NMR of Complex **9a-*d*$_2$** compared with that of **9a**.

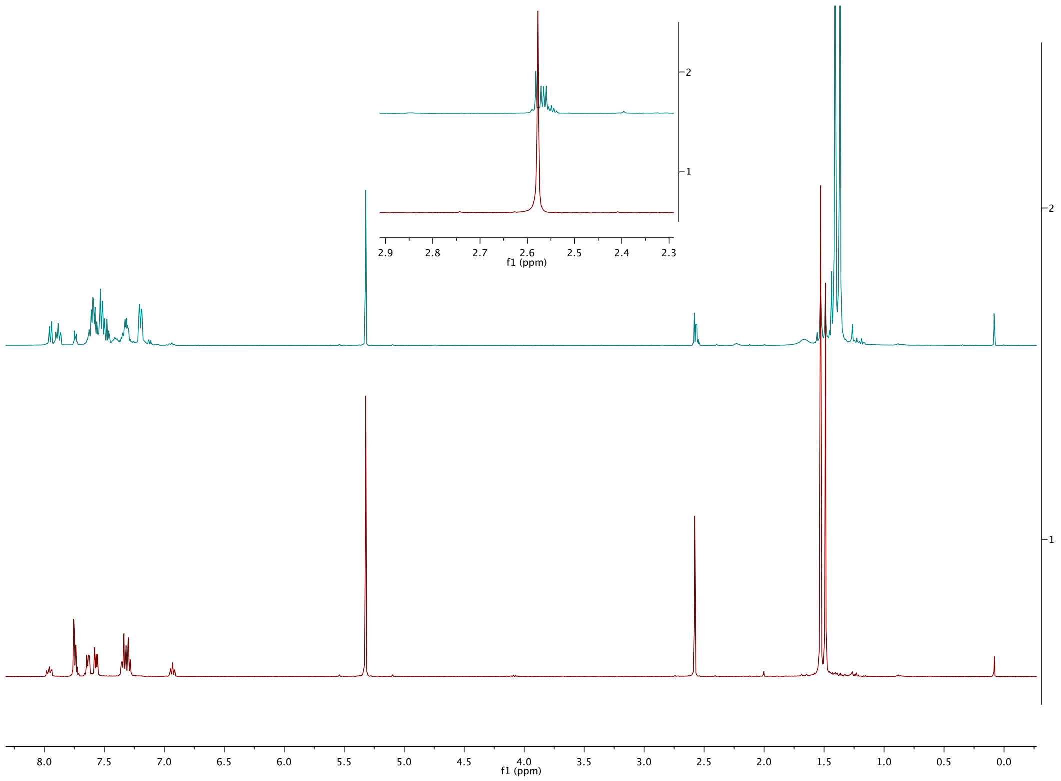

1,5-Disubstituted tetrazoles.

5-Methyl-1-phenyl-1*H*-tetrazole (8a).

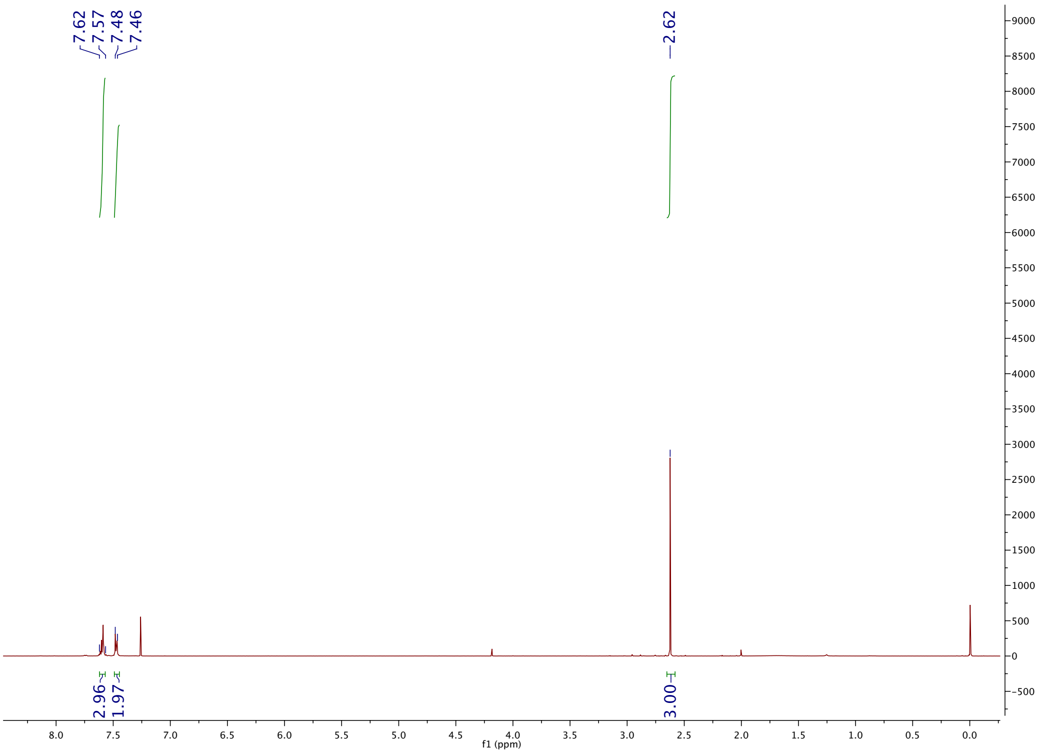

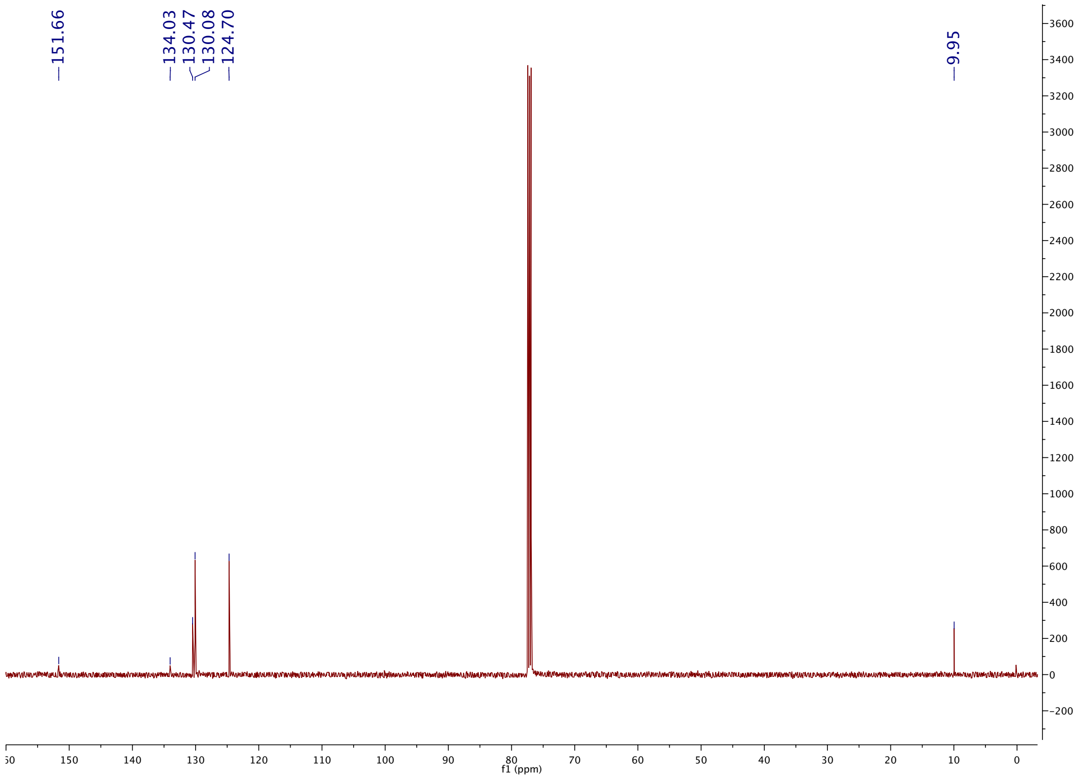

5-Methyl-1-(*p*-tolyl)-1*H*-tetrazole (8b).

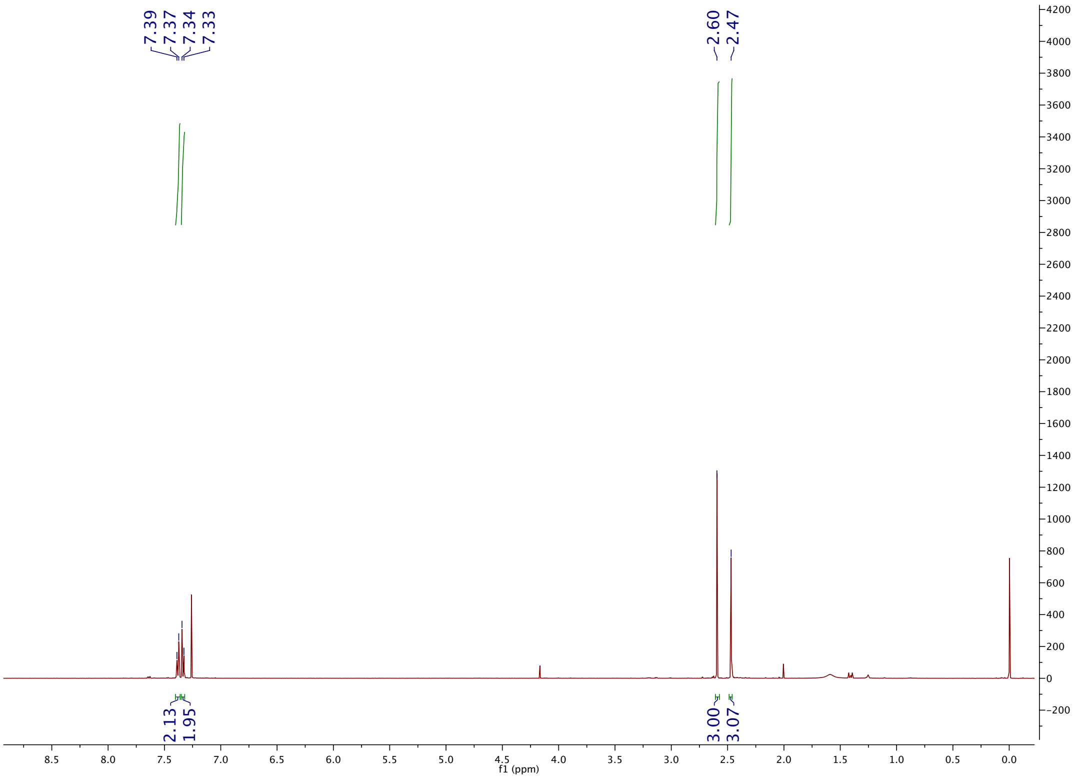

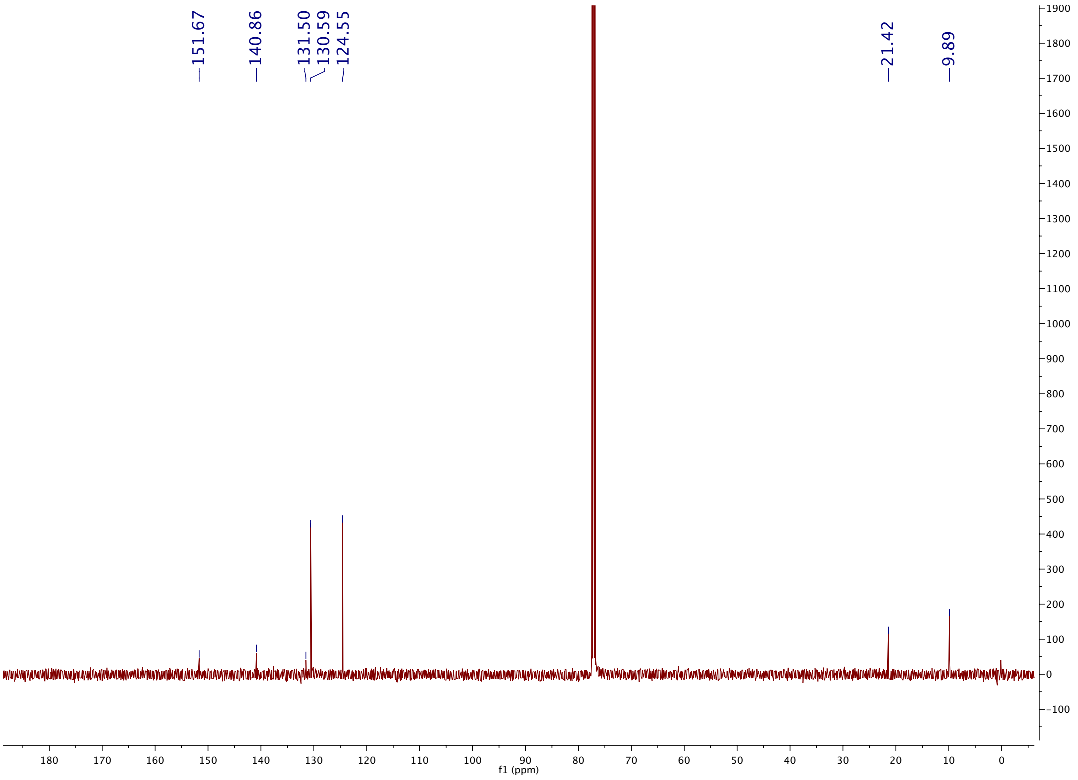

1-(4-Methoxyphenyl)-5-methyl-1*H*-tetrazole (8d).

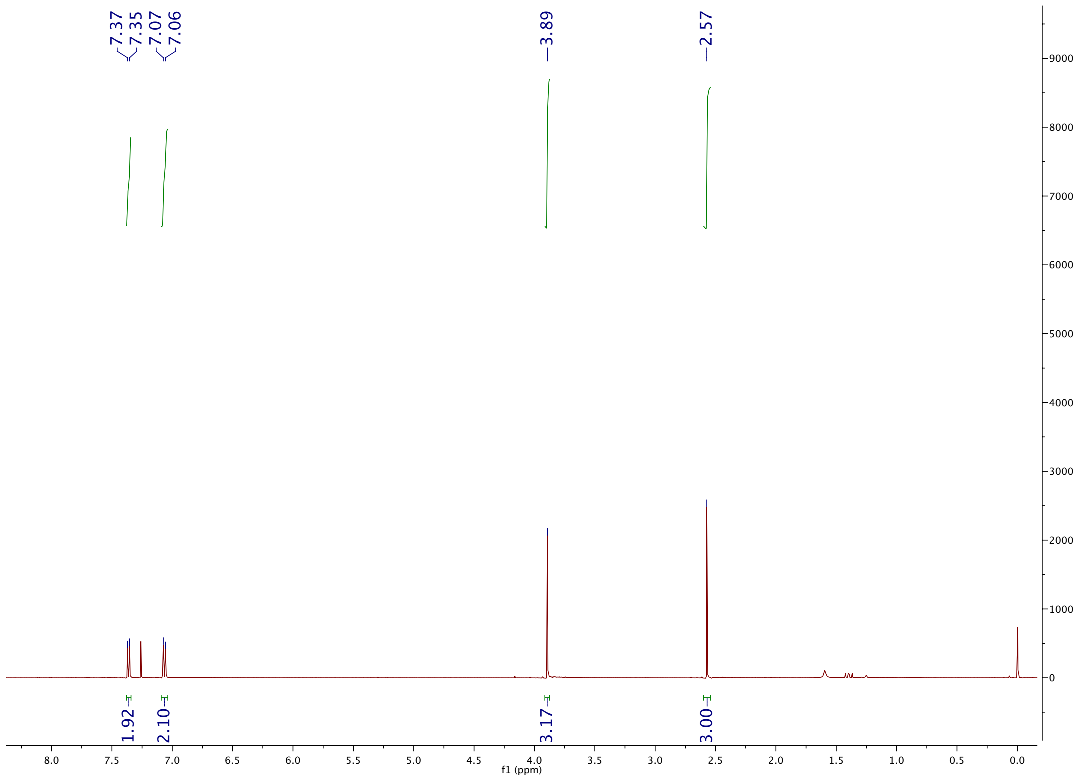

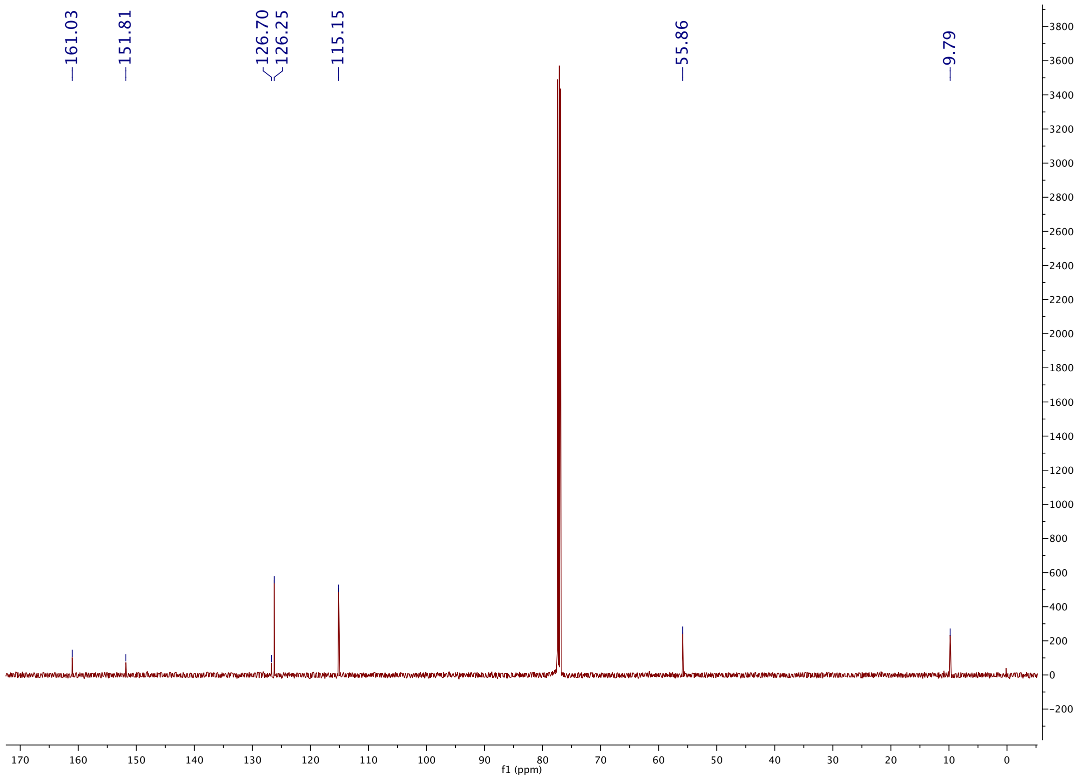

1-(3-Methoxyphenyl)-5-methyl-1*H*-tetrazole (8e).

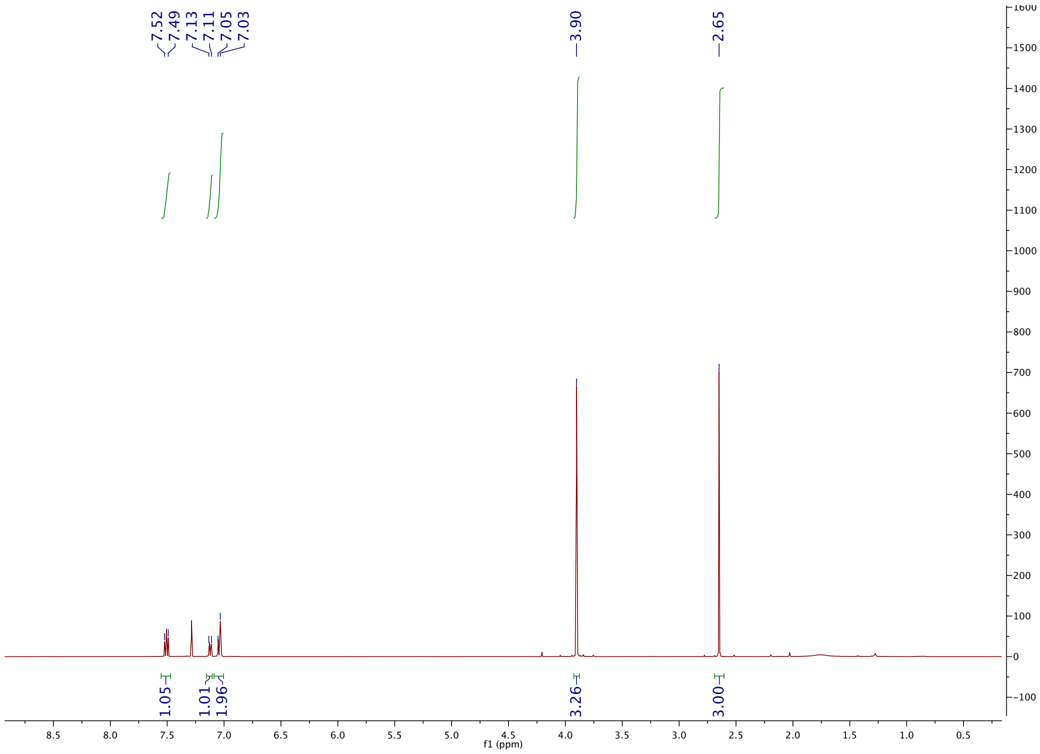

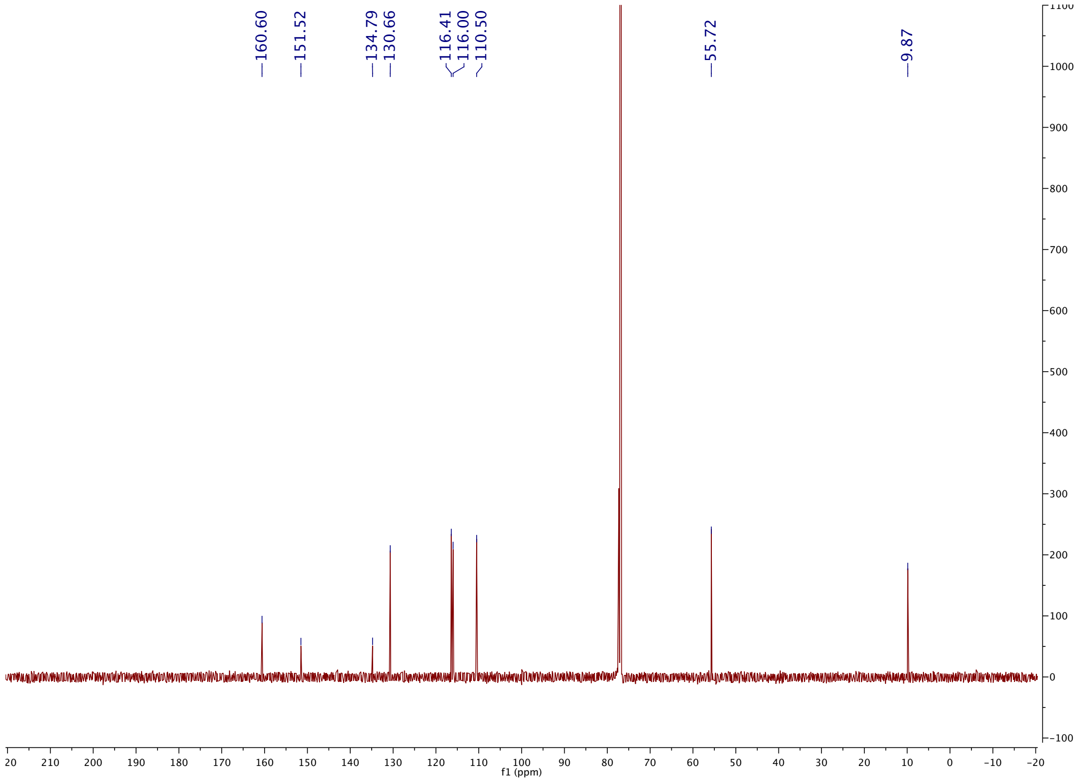

1-(2-Methoxyphenyl)-5-methyl-1*H*-tetrazole (8f).

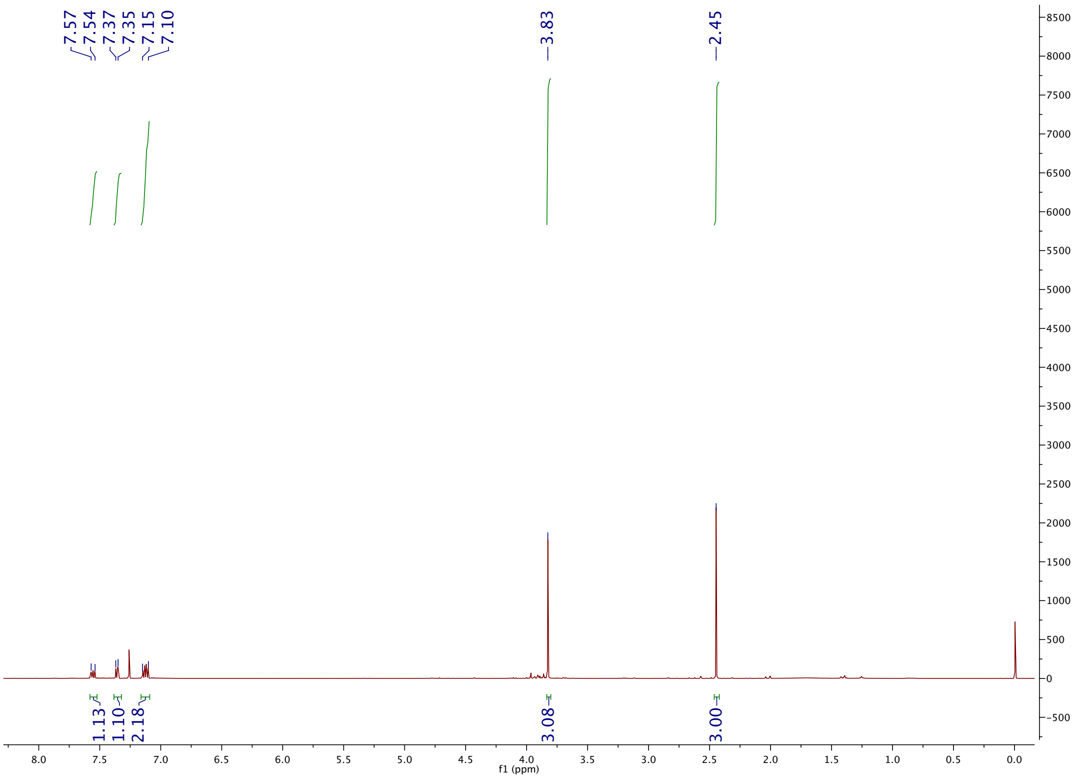

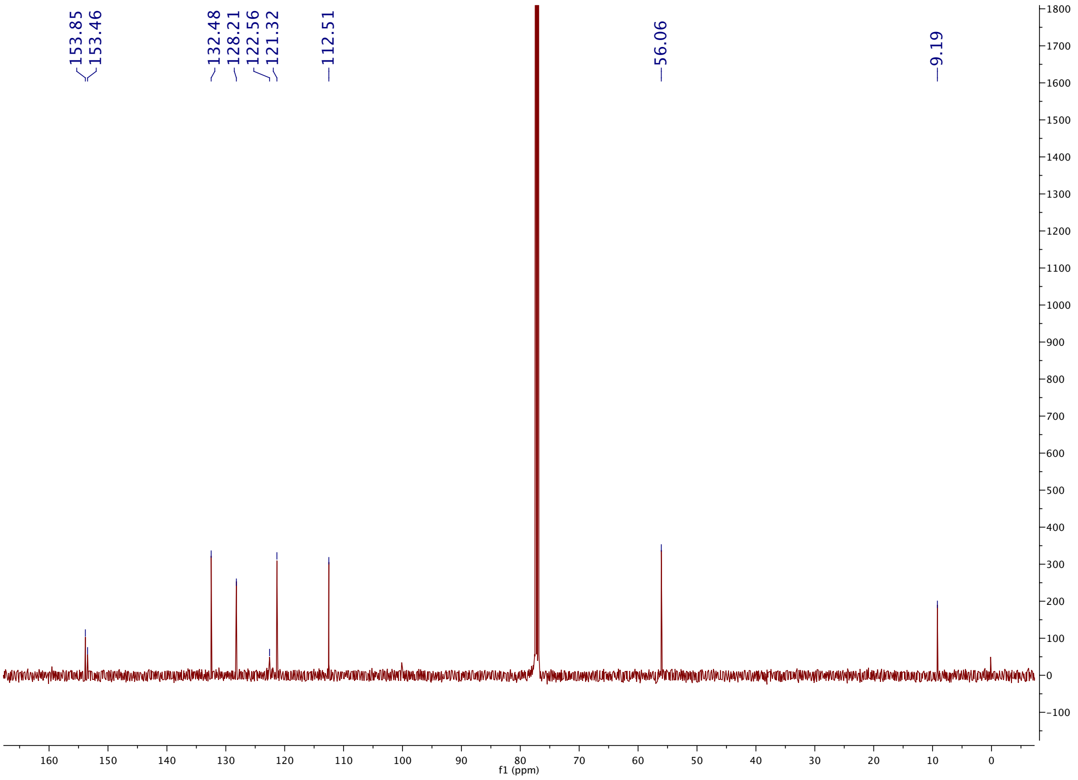

1-(4-Bromophenyl)-5-methyl-1*H*-tetrazole (8g).

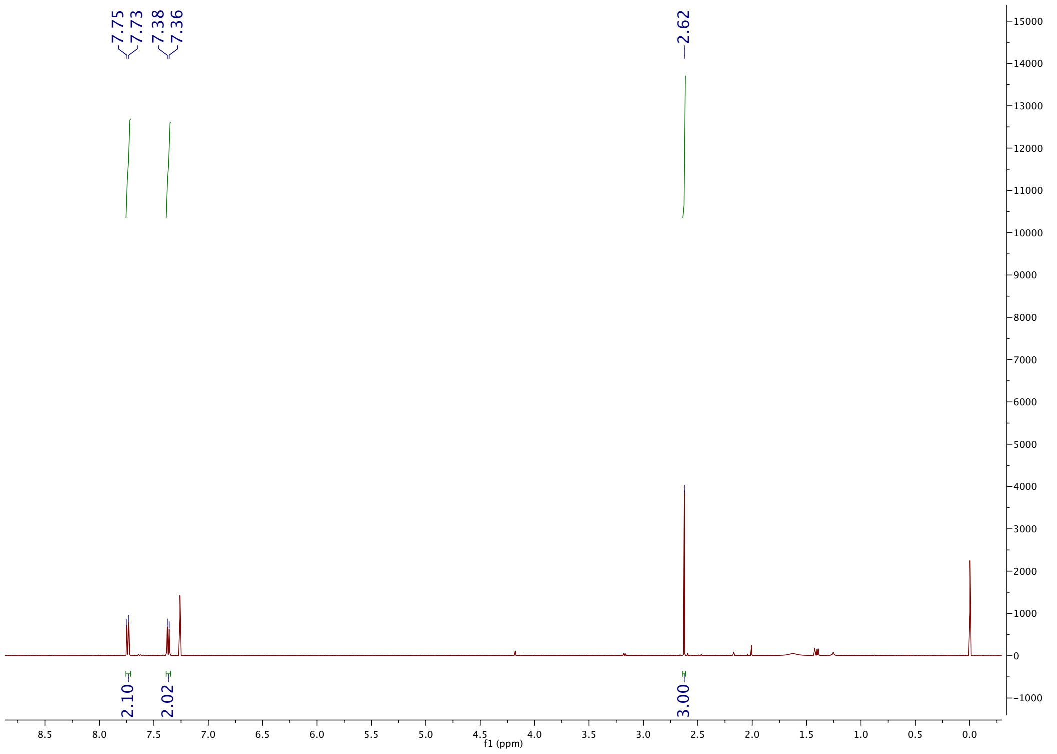

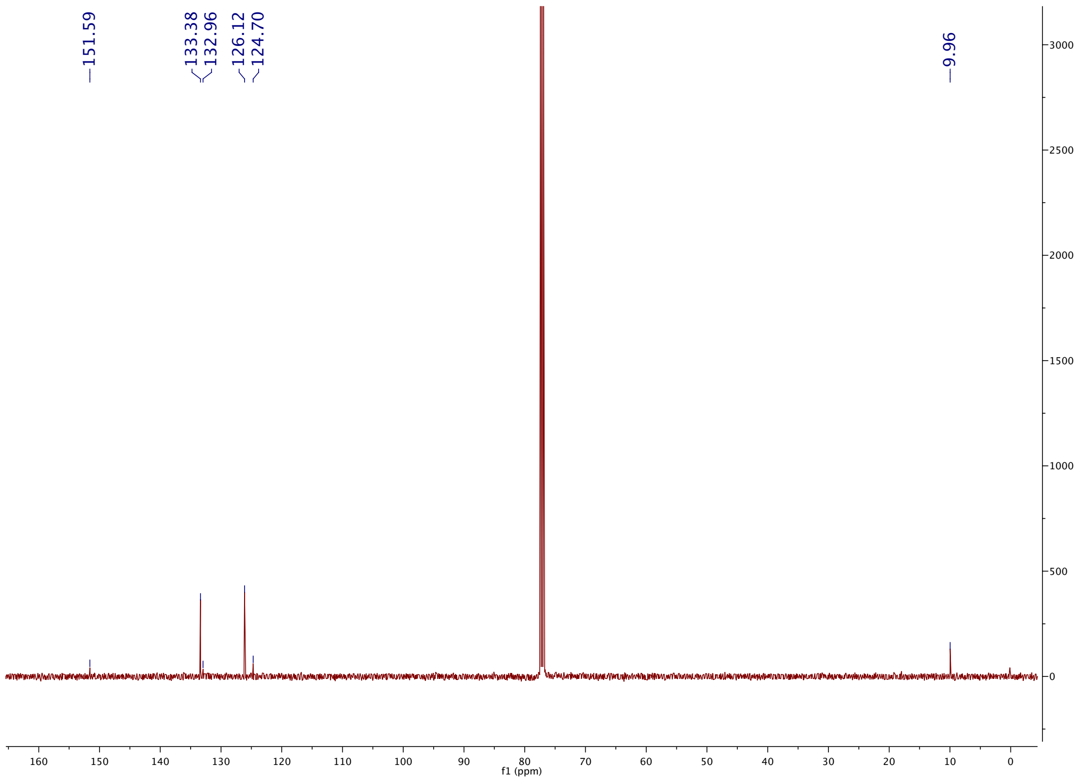

1-(4-(*tert*-Butyl)phenyl)-5-methyl-1*H*-tetrazole (8h).

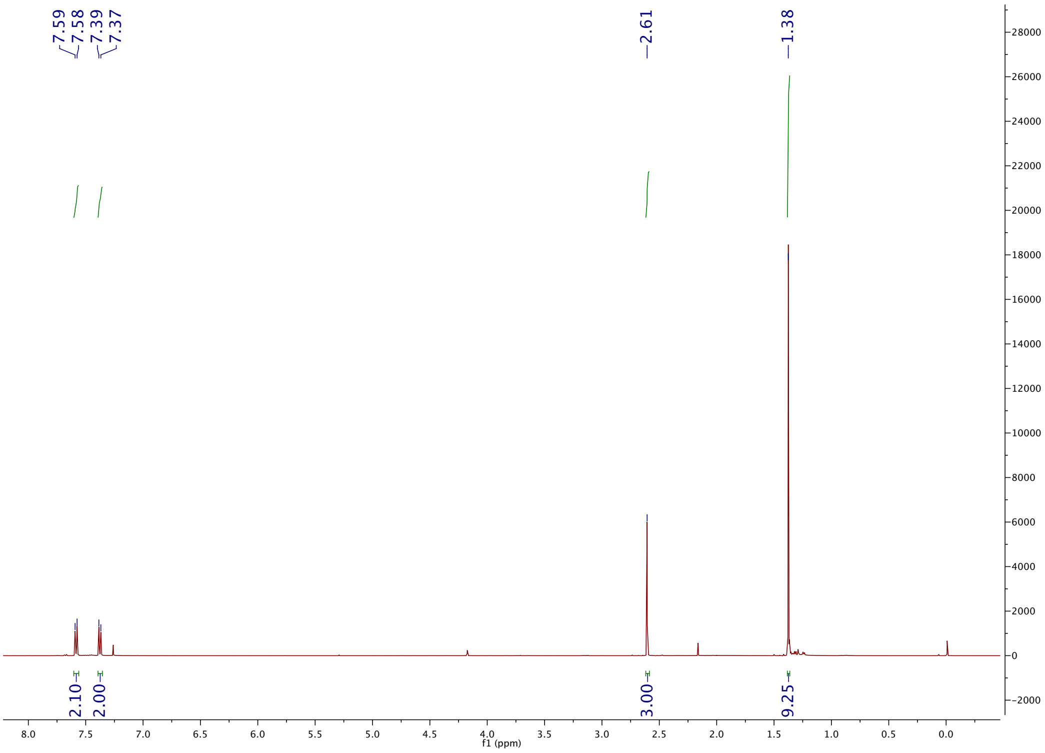

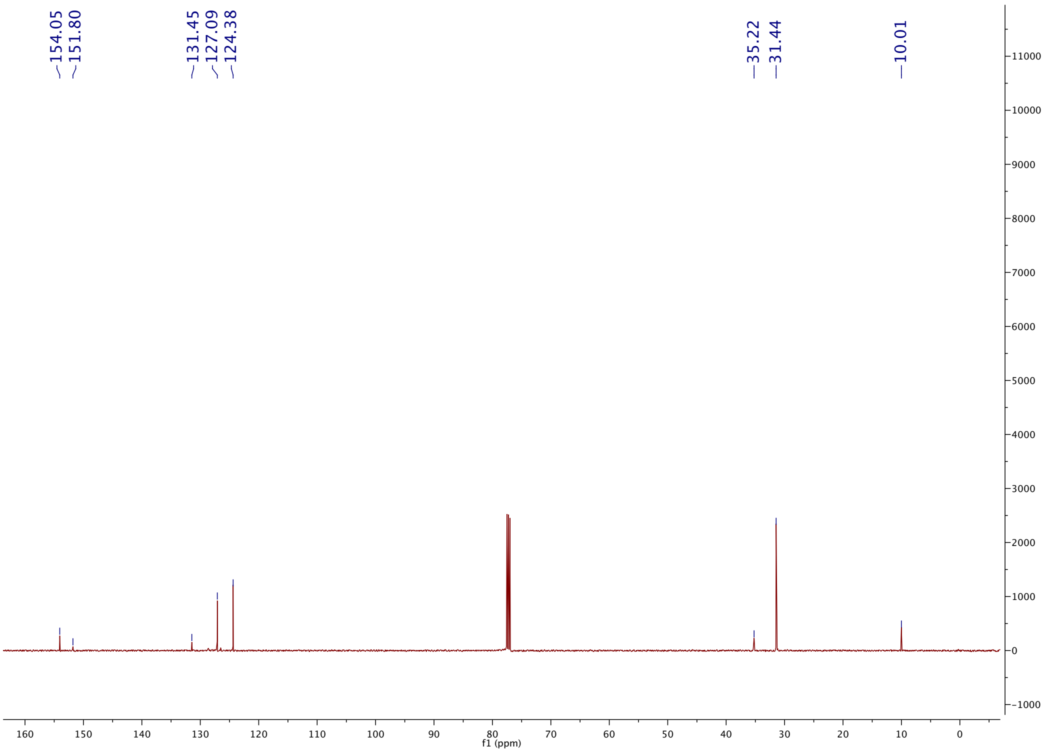

1-([1,1'-Biphenyl]-4-yl)-5-methyl-1*H*-tetrazole (8i).

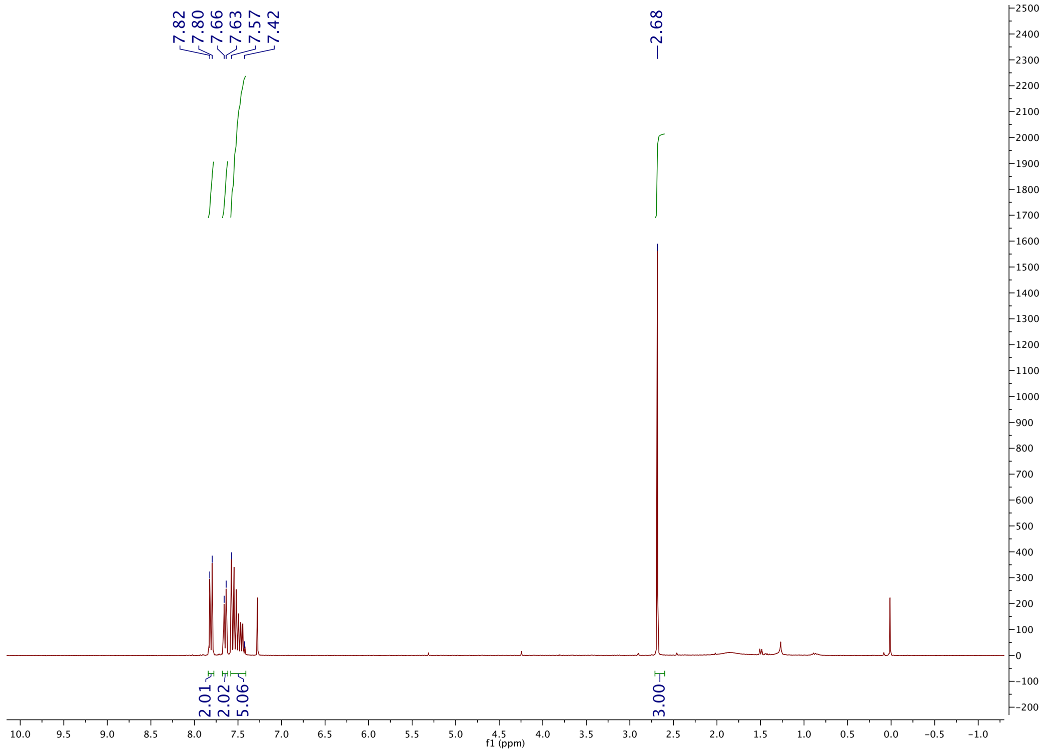

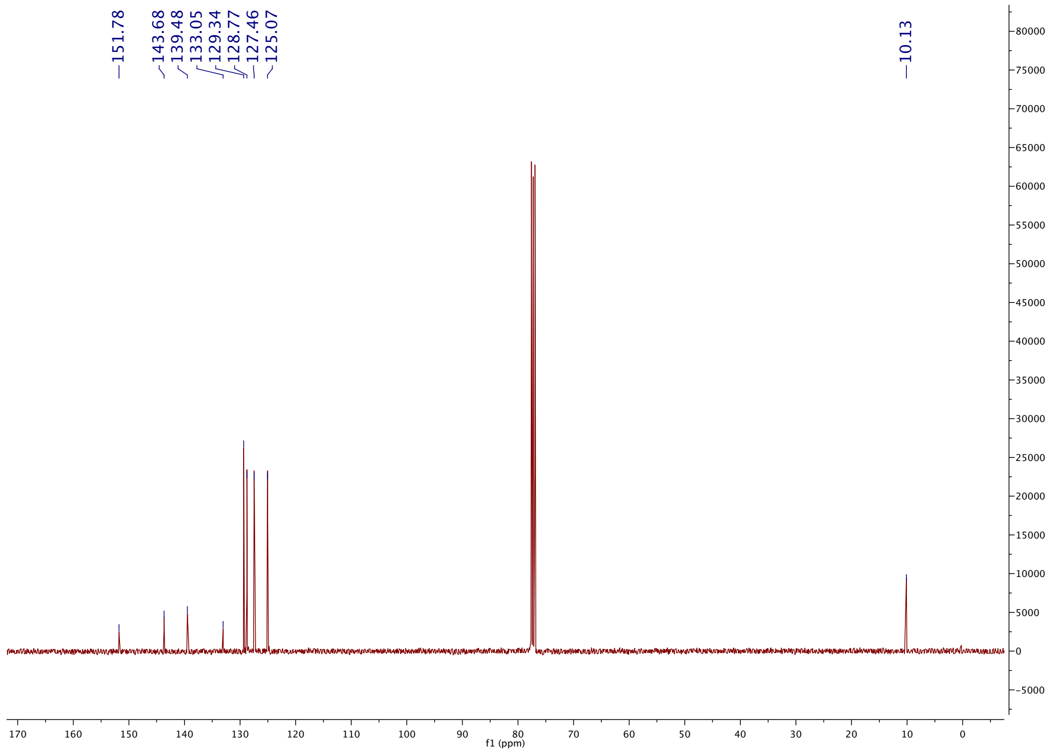

5-Methyl-1-(naphthalen-1-yl)-1*H*-tetrazole (8j).

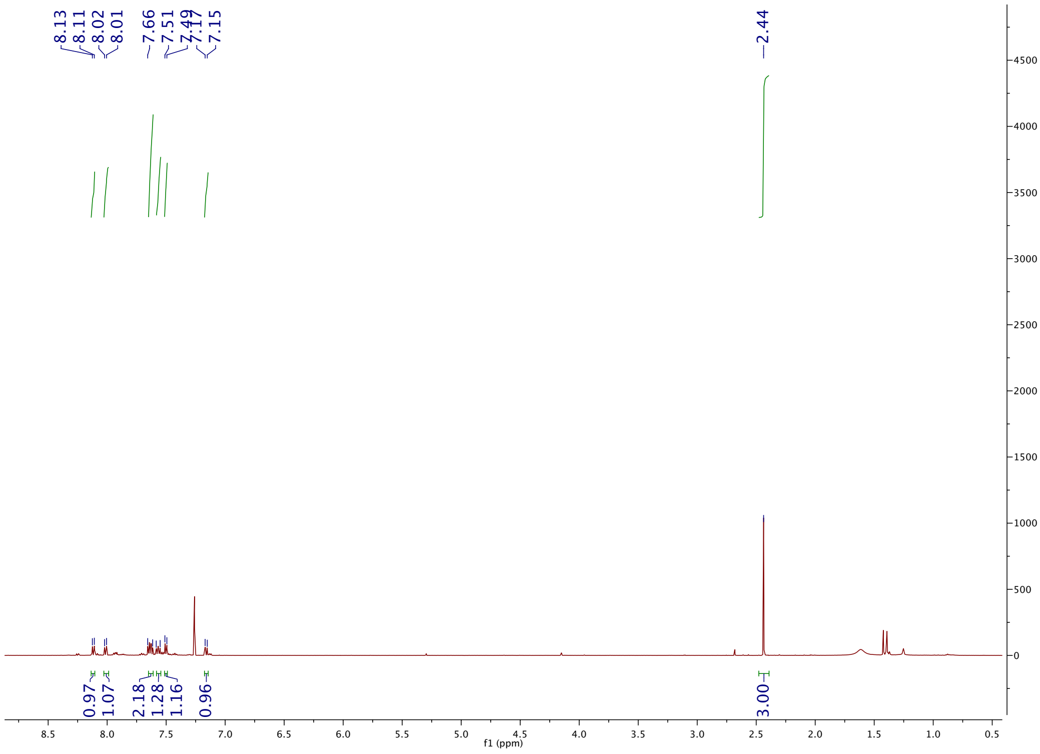

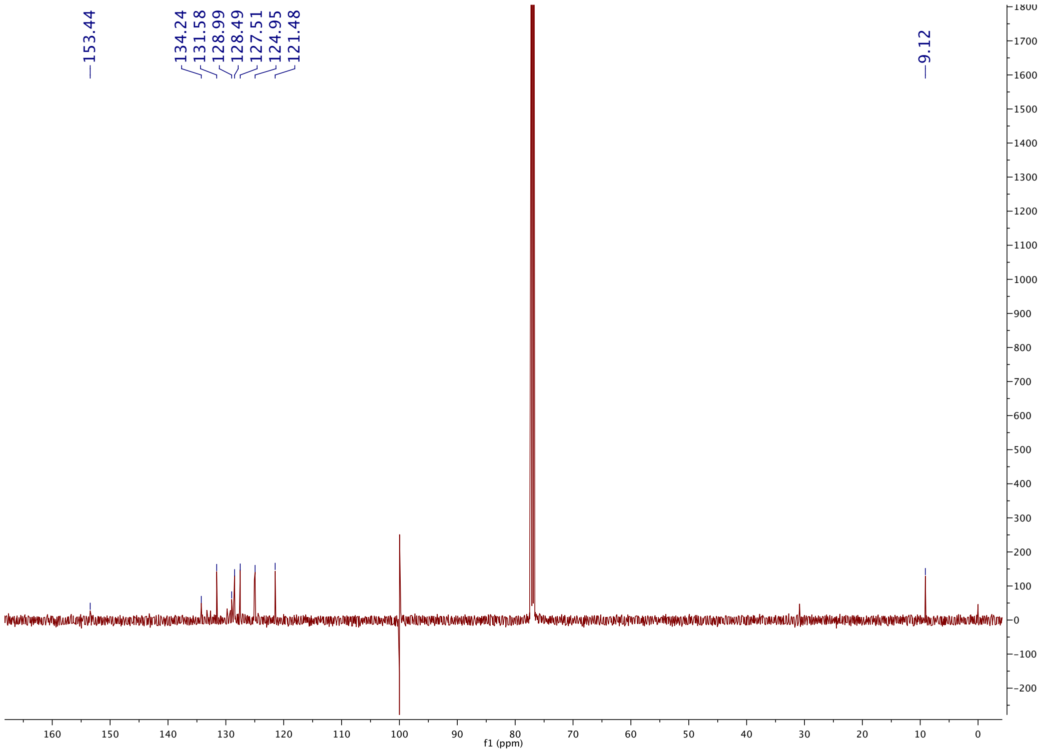

5-Methyl-1-(4-(trifluoromethyl)phenyl)-1*H*-tetrazole (8k).

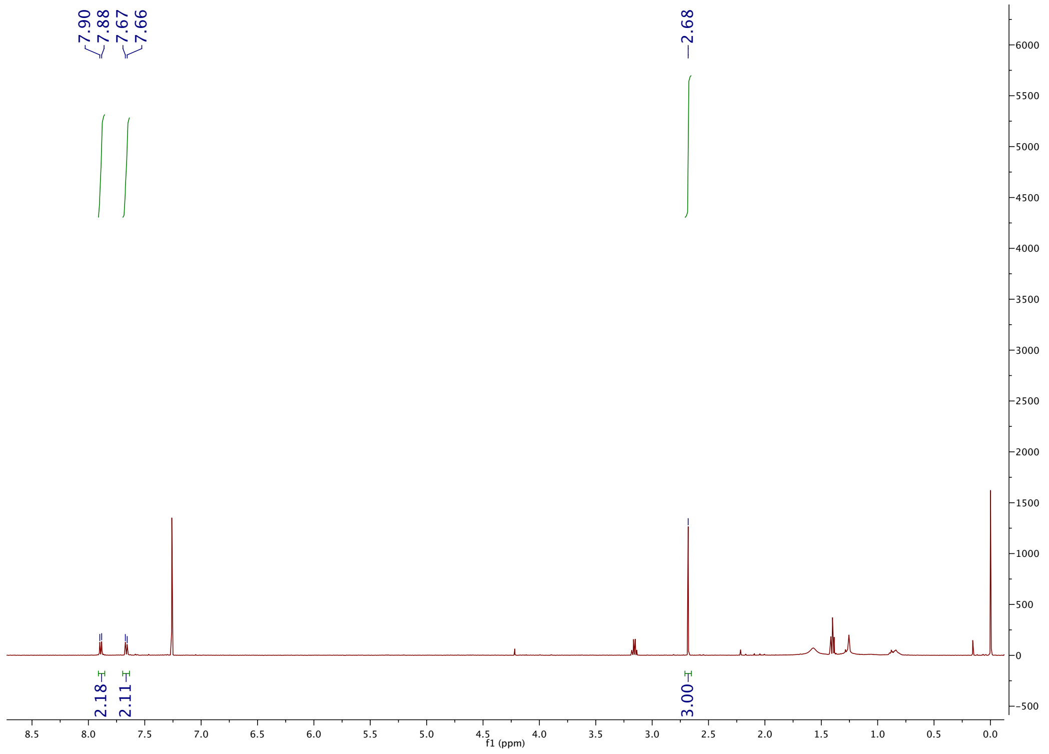

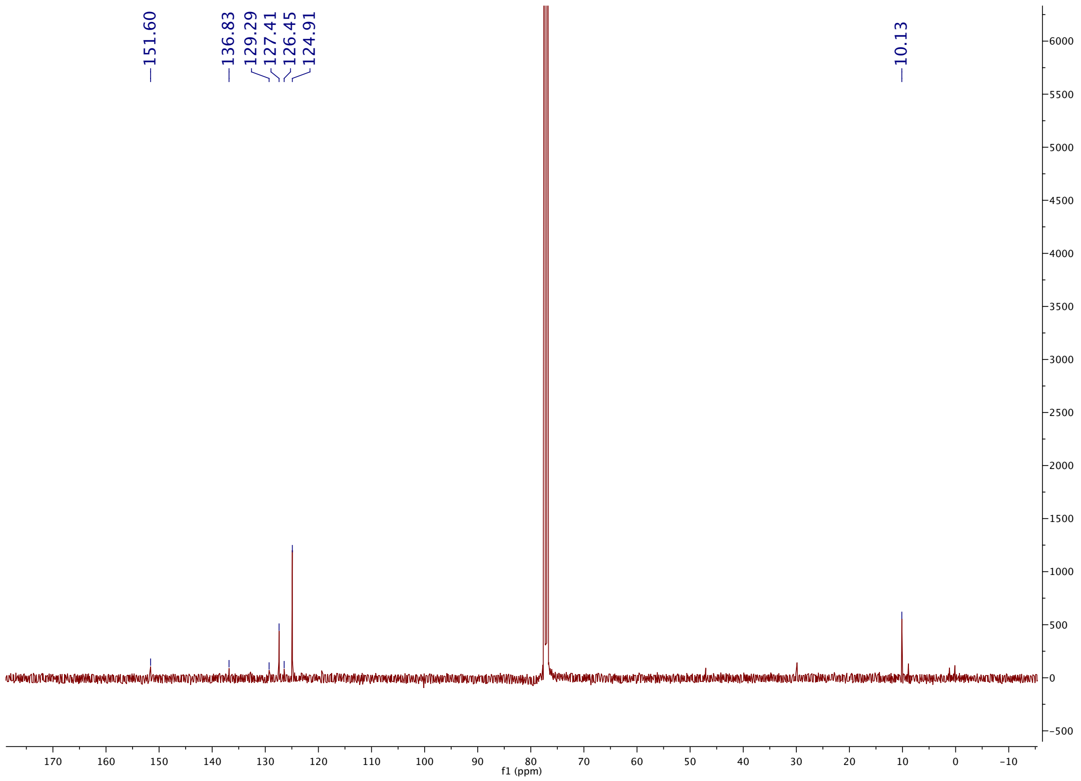

5-Methyl-1-(thiophen-3-yl)-1*H*-tetrazole (8l).

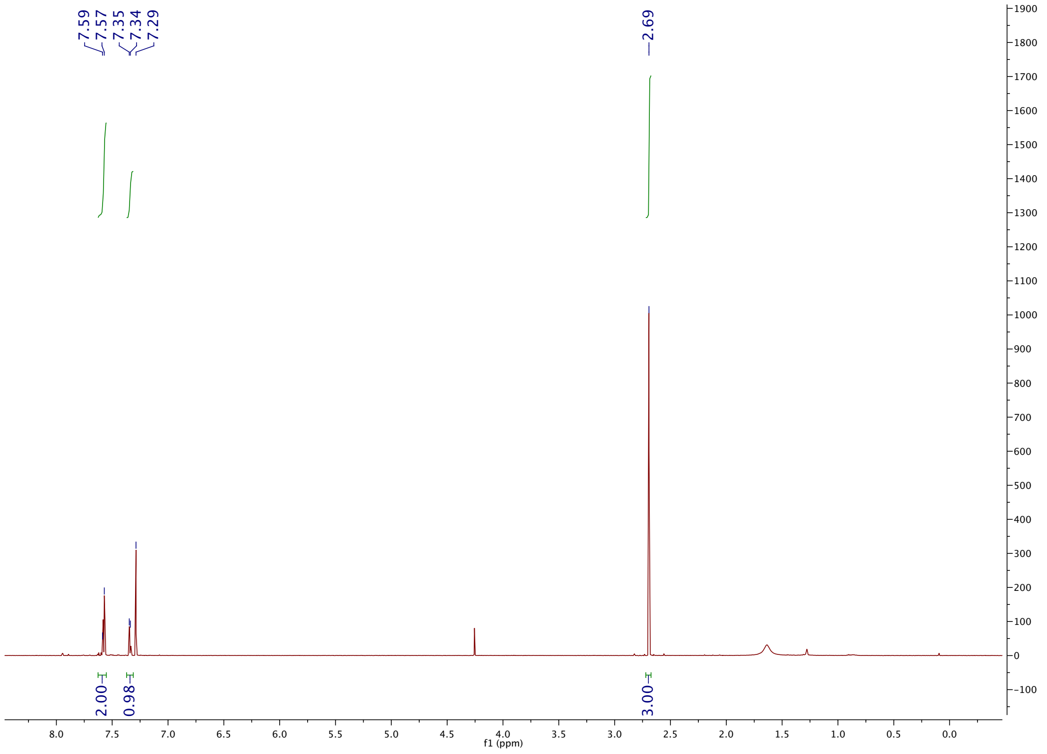

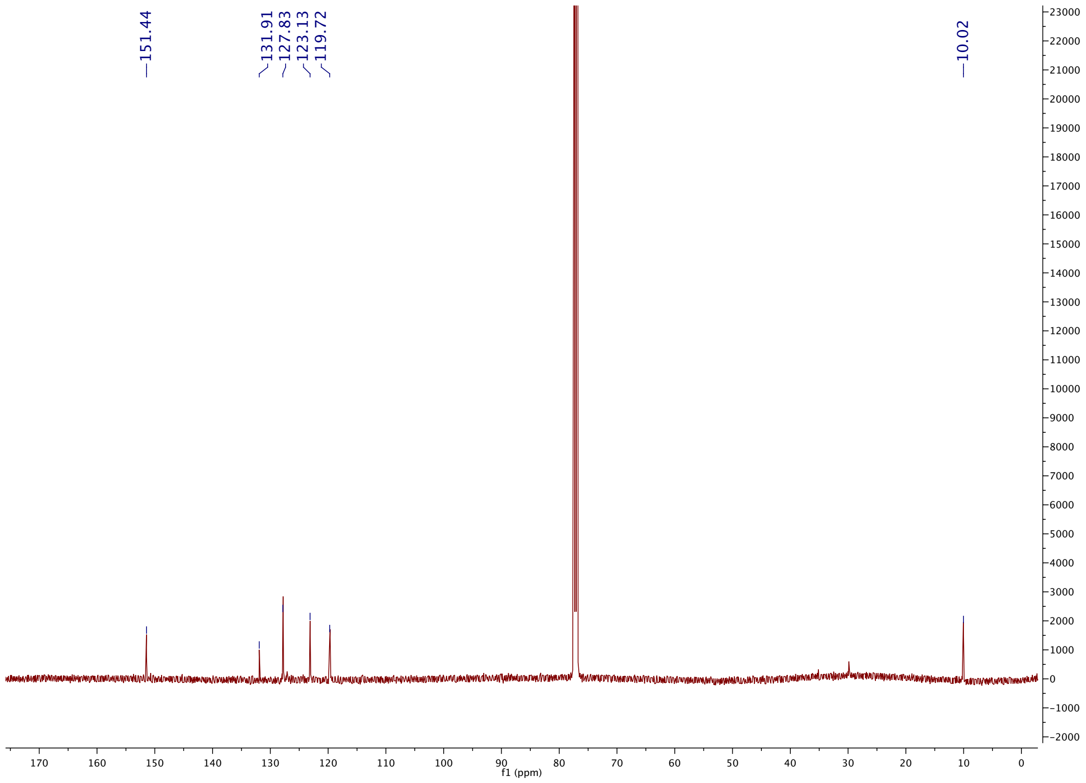

1-Cyclohexyl-5-methyl-1*H*-tetrazole (8m).

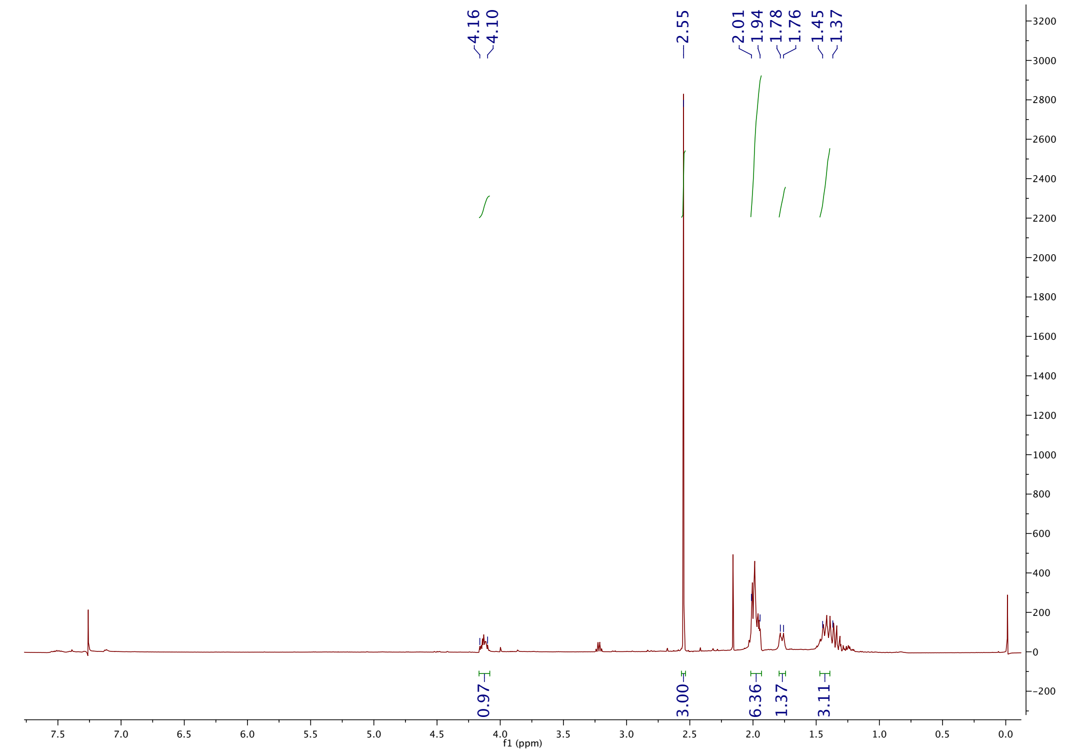

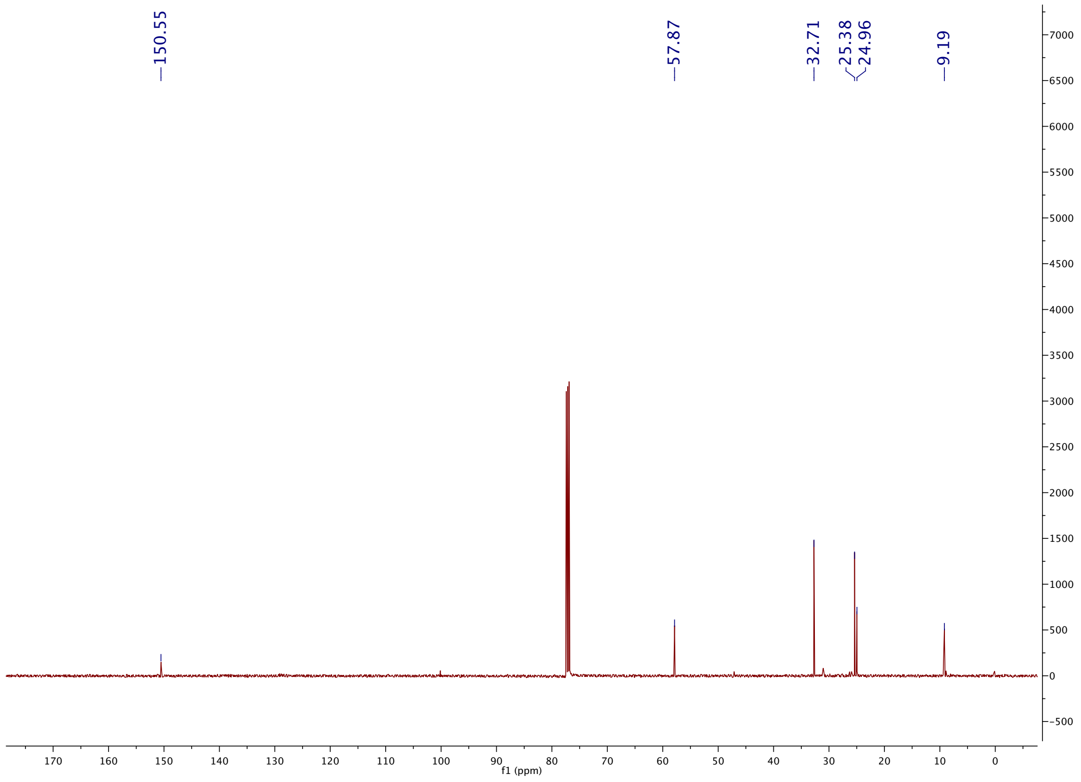

5-Methyl-1-propyl-1*H*-tetrazole and 1-methyl-5-propyl-1*H*-tetrazole (8n, 8n').

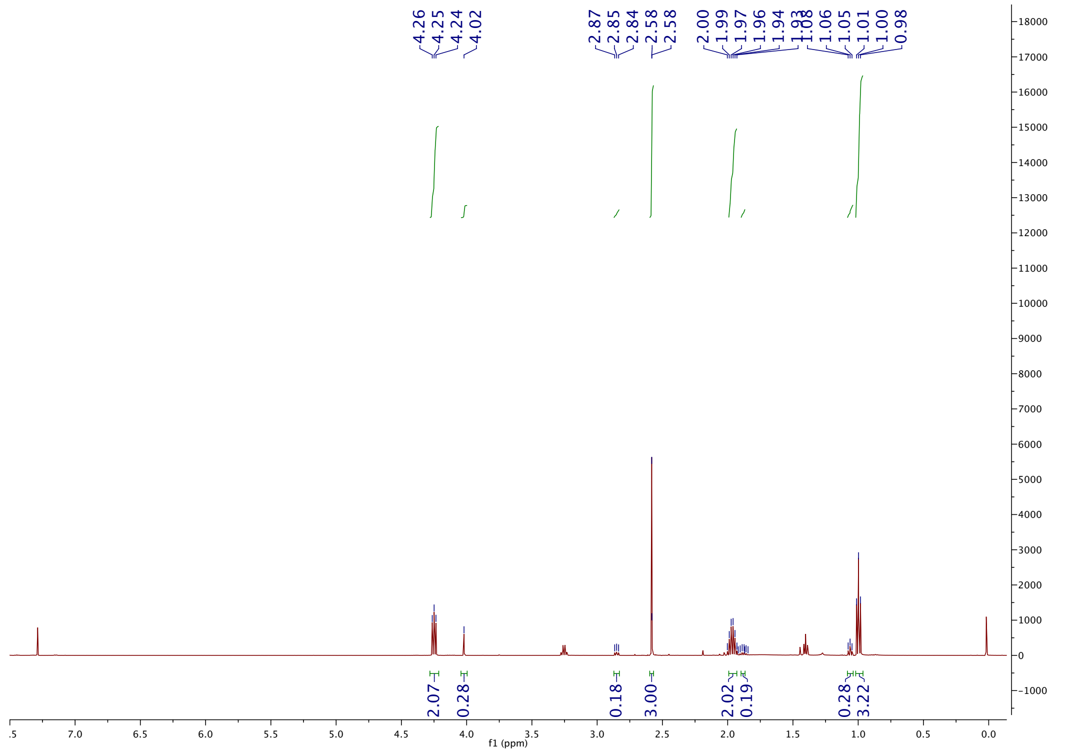

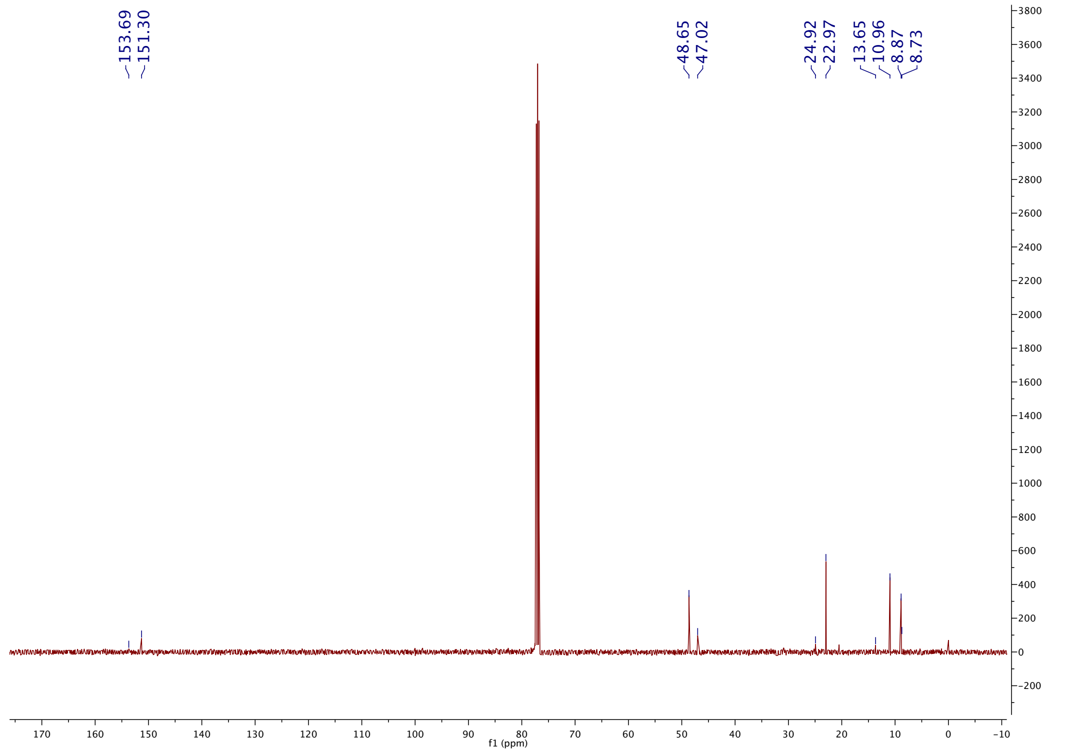

1-Cyclopropyl-5-methyl-1*H*-tetrazole and 5-cyclopropyl-1-methyl-1*H*-tetrazole (8o, 8o').

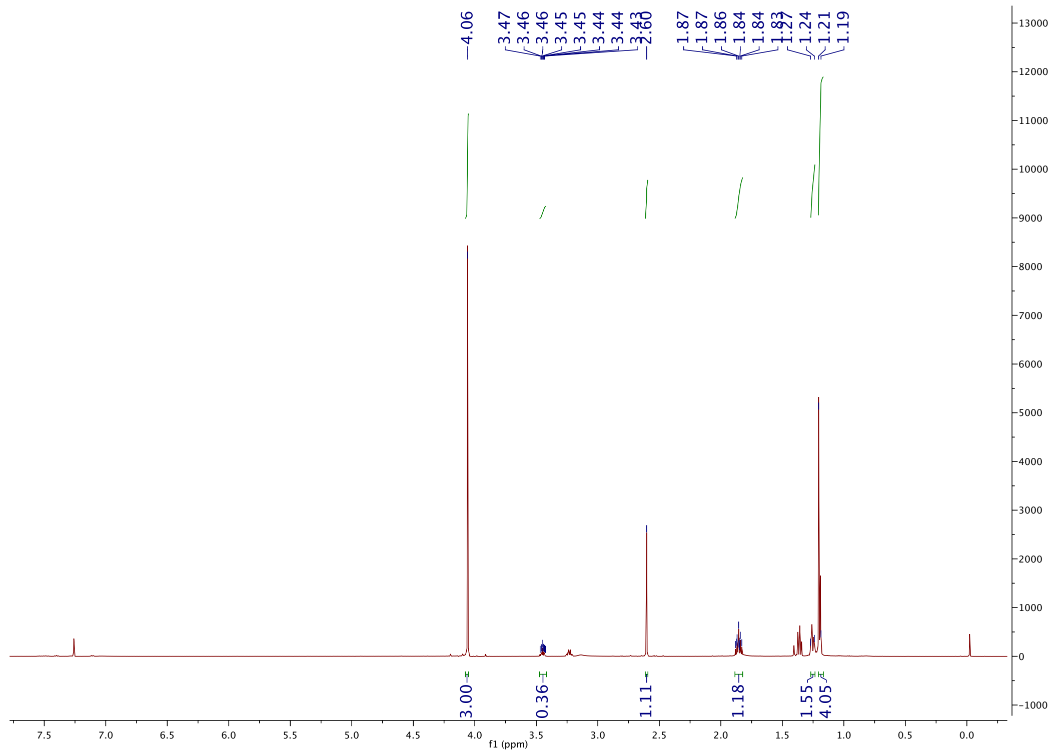

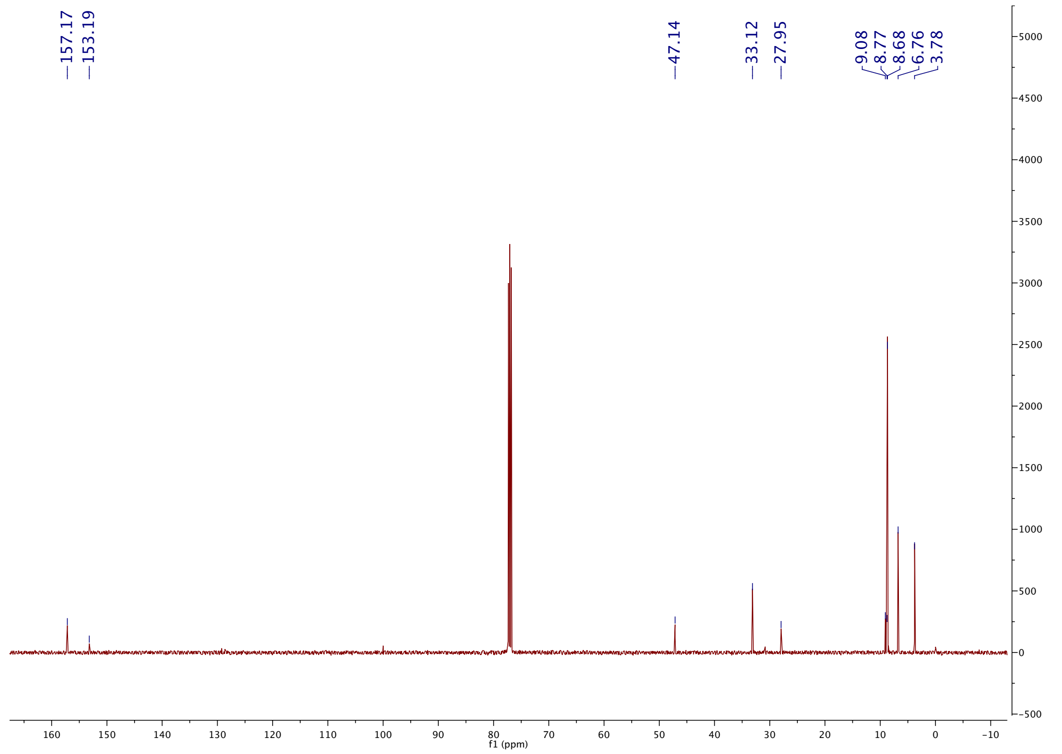

1-(1-Azidovinyl)-4-nitrobenzene (5c).

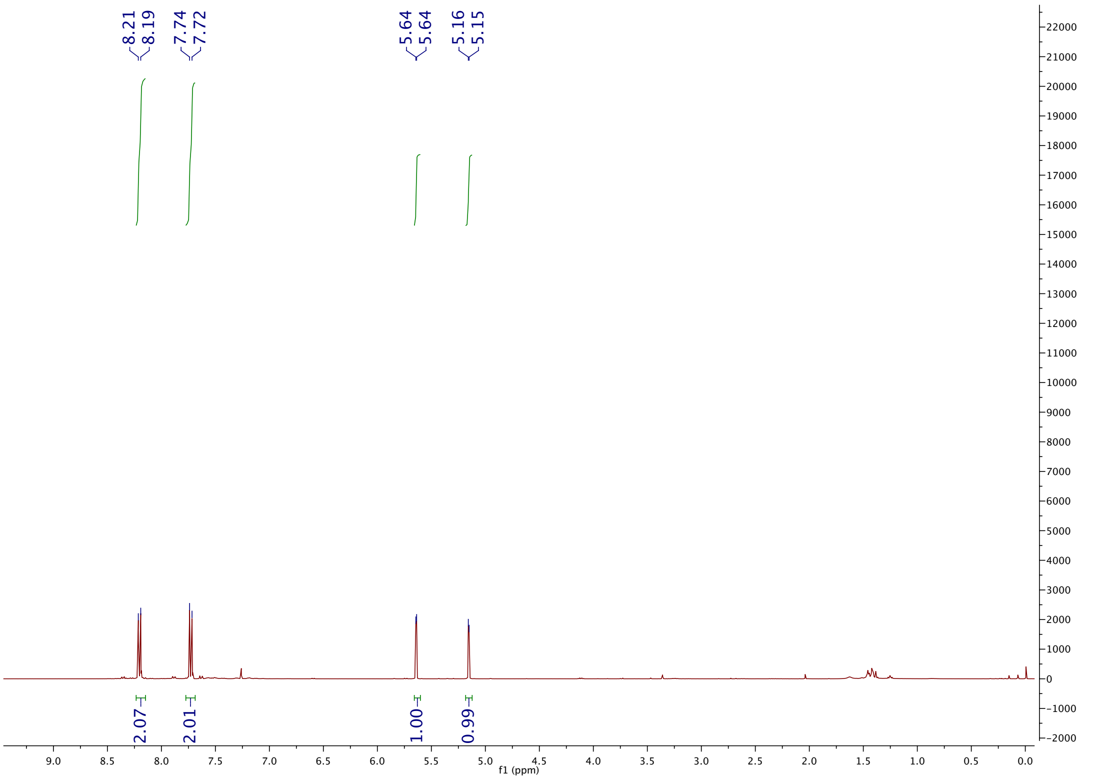

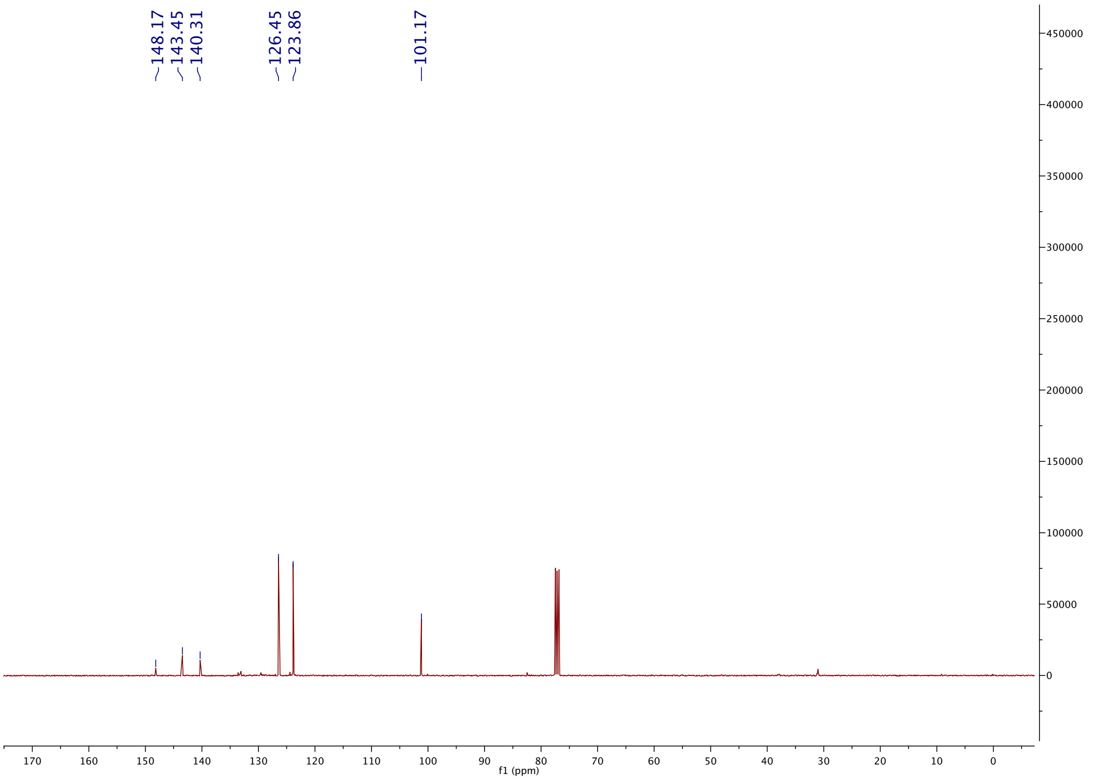

X-ray Crystal Structure Determinations.

Crystal data for complex 9c.

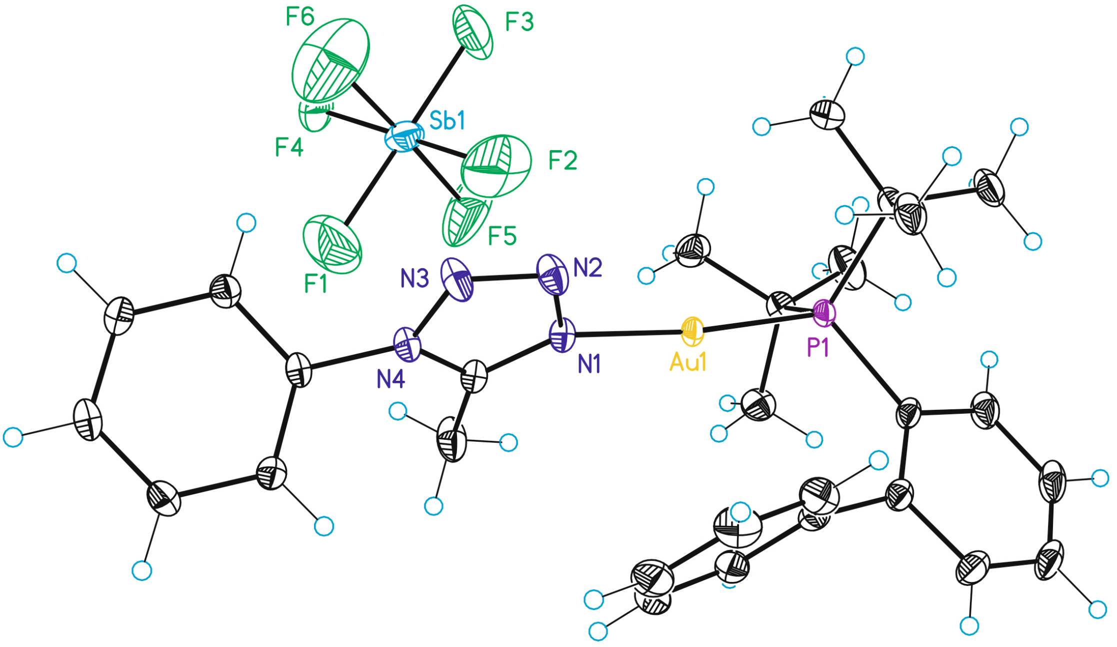

Table 1. Crystal data and structure refinement for mo_MG11F_0m.

Identification code	mo_MG11F_0m	
Empirical formula	C14 H17.50 Au0.50 F3 N2 P0.50 Sb0.50	
Formula weight	445.64	
Temperature	100(2) K	
Wavelength	0.71073 Å	
Crystal system	Monoclinic	
Space group	C2/c	
Unit cell dimensions	a = 26.138(2) Å	a= 90.00 °.
	b = 9.8567(8) Å	b = 92.197(2) °.
	c = 24.3524(19) Å	g = 90.00 °.
Volume	6269.5(9) Å^3	
Z	16	
Density (calculated)	1.889 Mg/m^3	
Absorption coefficient	5.648 mm^{-1}	
F(000)	3440	
Crystal size	0.15 x 0.10 x 0.10 mm^3	
Theta range for data collection	1.56 to 30.50 °.	
Index ranges	-27 <=h<=36 ,-12 <=k<=13 ,-34 <=l<=33	
Reflections collected	42491	

Independent reflections	8608 [R(int) = 0.0201]
Completeness to theta =30.50 °	90.100006%
Absorption correction	Empirical
Max. and min. transmission	0.6020 and 0.4846
Refinement method	Full-matrix least-squares on F^2
Data / restraints / parameters	8608 / 147 / 440
Goodness-of-fit on F^2	0.995
Final R indices [I>2sigma(I)]	R1 = 0.0171 , wR2 = 0.0455
R indices (all data)	R1 = 0.0185 , wR2 = 0.0462
Largest diff. peak and hole	0.843 and -1.155 e.Å^{-3}

Crystal data for complex 9b.

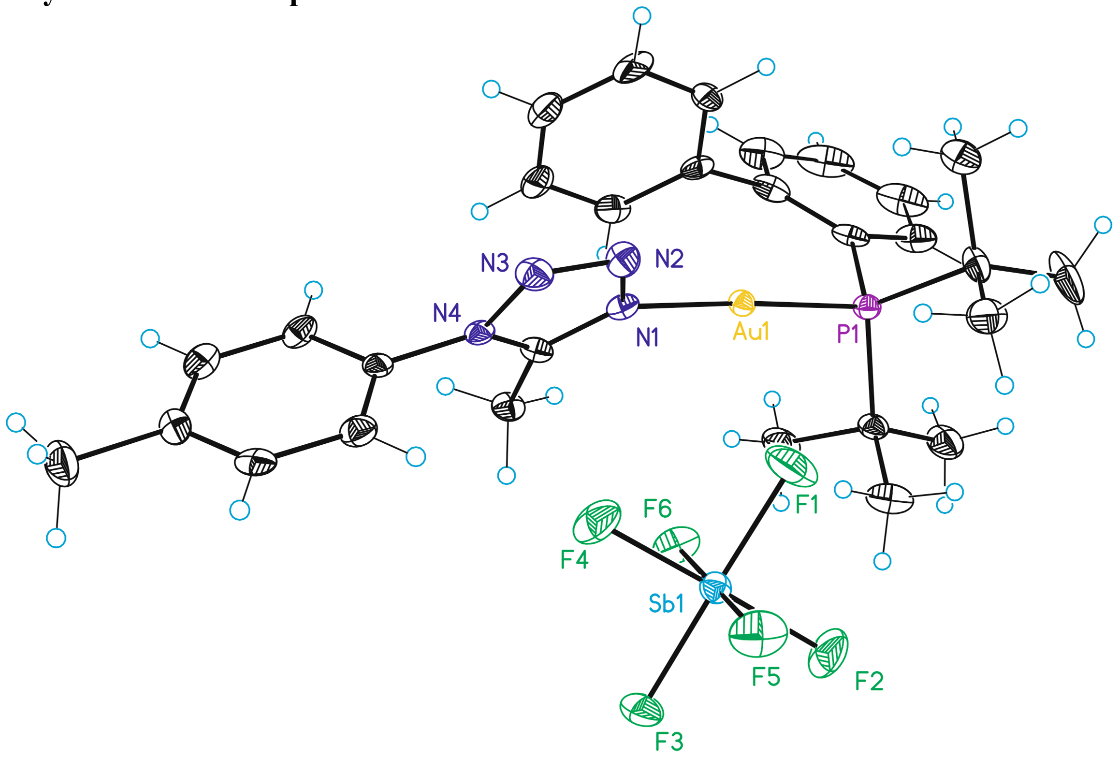

Table 1. Crystal data and structure refinement for mo_MG016F_0m.

Identification code	mo_MG016F_0m	
Empirical formula	C31 H40 Au F6 N4 O P Sb	
Formula weight	948.36	
Temperature	100(2) K	
Wavelength	0.71073 Å	
Crystal system	Triclinic	
Space group	P-1	
Unit cell dimensions	a = 10.1685(8) Å	〈= 85.701(3) °.
	b = 13.3982(11) Å	® = 68.859(3) °.
	c = 13.7328(12) Å	© = 77.800(3) °.
Volume	1705.6(2) $Å^3$	
Z	2	
Density (calculated)	1.847 Mg/m^3	
Absorption coefficient	5.198 mm^{-1}	
F(000)	922	
Crystal size	0.10 x 0.08 x 0.02 mm^3	
Theta range for data collection	1.55 to 26.48 °.	
Index ranges	-12 <=h<=11 ,-16 <=k<=16 ,-17 <=l<=17	
Reflections collected	16630	
Independent reflections	6983 [R(int) = 0.0587]	

Completeness to theta =26.48 °	99.0%
Absorption correction	Empirical
Max. and min. transmission	0.9032 and 0.6245
Refinement method	Full-matrix least-squares on F^2
Data / restraints / parameters	6983 / 42 / 442
Goodness-of-fit on F^2	0.989
Final R indices [I>2sigma(I)]	R1 = 0.0363 , wR2 = 0.0656
R indices (all data)	R1 = 0.0571 , wR2 = 0.0722
Largest diff. peak and hole	1.312 and -1.494 e.Å^{-3}

Crystal data for complex 9c.

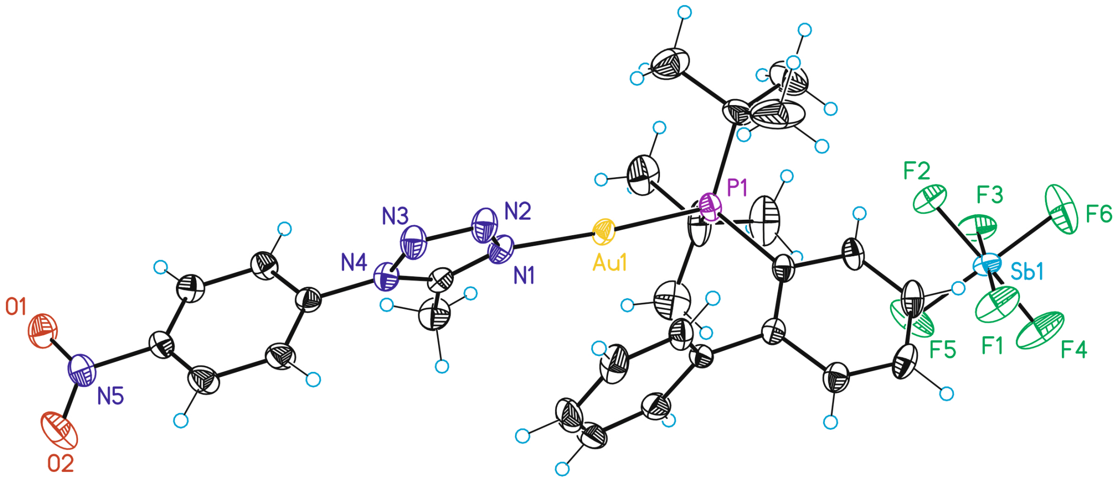

Table 1. Crystal data and structure refinement for mo_mg019F_0m.

Identification code	mo_mg019F_0m	
Empirical formula	C28 H34 Au F6 N5 O2 P Sb	
Formula weight	936.29	
Temperature	100(2) K	
Wavelength	0.71073 Å	
Crystal system	Triclinic	
Space group	P-1	
Unit cell dimensions	a = 9.9683(6) Å	⟨= 111.409(2) °.
	b = 12.4112(8) Å	® = 107.903(2) °.
	c = 15.3013(9) Å	© = 92.245(2) °.
Volume	1652.42(18) $Å^3$	
Z	2	
Density (calculated)	1.882 Mg/m^3	
Absorption coefficient	5.367 mm^{-1}	
F(000)	904	
Crystal size	0.15 x 0.15 x 0.03 mm^3	
Theta range for data collection	1.79 to 30.36 °.	
Index ranges	-13 <=h<=13 ,-17 <=k<=16 ,-21 <=l<=20	
Reflections collected	19645	
Independent reflections	8644 [R(int) = 0.0210]	
Completeness to theta =30.36 °	86.9%	
Absorption correction	Empirical	
Max. and min. transmission	0.8556 and 0.4999	

Refinement method	Full-matrix least-squares on F^2
Data / restraints / parameters	8644 / 0 / 404
Goodness-of-fit on F^2	1.071
Final R indices [I>2sigma(I)]	R1 = 0.0425 , wR2 = 0.1074
R indices (all data)	R1 = 0.0462 , wR2 = 0.1126
Largest diff. peak and hole	5.663 and -1.190 e.Å^{-3}
